# Relationship between Vaccine Application and Climate Factors in Sheep and Goat Farms in Greece

**DOI:** 10.3390/vaccines11040797

**Published:** 2023-04-04

**Authors:** Eleni I. Katsarou, George C. Fthenakis

**Affiliations:** Veterinary Faculty, University of Thessaly, 43100 Karditsa, Greece

**Keywords:** agricultural environment, climate, climate change, health management, goat, sheep, vaccine

## Abstract

The objectives of the present study were (a) to evaluate the importance of climate-related variables in the vaccination patterns applied in sheep and goat farms in Greece and (b) to assess potential interactions between these factors and previously established important health management- and human resources-related factors applied in the farms. Vaccination patterns against chlamydial abortion, clostridial infections, contagious agalactia, contagious ecthyma, foot-rot, paratuberculosis, pneumonia or staphylococcal mastitis were assessed. Climatic variables (2010–2019 and 2018–2019) were obtained for 444 locations with small ruminant farms throughout Greece. Patterns of vaccine administration in the farms were obtained through interviews with farmers. The following nine outcomes were considered: ‘vaccination against chlamydial abortion’, ‘vaccination against clostridial infections’, ‘vaccination against contagious agalactia’, ‘vaccination against contagious ecthyma’, ‘vaccination against foot-rot’, ‘vaccination against paratuberculosis’, ‘vaccination against bacterial pneumonia’, ‘vaccination against staphylococcal mastitis’ and ‘total number of optional vaccines administered’. Univariable and multivariable analyses were first performed to establish associations of each of the above outcomes with climatic variables. Then, the same approach was employed to assess the importance of climatic variables against health management- and human resources-related factors in the administration of vaccines in the farms of the study. Climatic variables had a higher association with vaccinations against infections in sheep flocks (26 associations) than in goat herds (9 associations) (*p* = 0.002) and in farms with semi-extensive or extensive management (32 associations) than in farms with intensive or semi-intensive management (8 associations) (*p* < 0.0001). In 26 cases (38.8% of all analyses evaluated), the climatic variables overshadowed the management- and human resources-related variables assessed as significant predictors for vaccination. In most cases, these referred to sheep flocks (nine cases) and farms with semi-extensive or extensive management (eight cases). For all eight infections, there were changes in the climatic variables found to be significant predictors from the 10-year dataset to the 2-year dataset. The results indicated that, in some cases, climate factors overshadowed factors traditionally considered for the formulation of vaccination programs. This points out the significance of taking into account climate conditions in the health management of small ruminant farms. Future studies can be focused on formulating vaccination programs in accordance with climate factors and also on setting the optimum season(s) for vaccination of animals based on the circulation of the pathogens, the risks for the development of diseases and the stage within the annual production cycle of the animals.

## 1. Introduction

Climate conditions (e.g., temperature, rainfall, relative humidity, wind speed) can play a role in the development of microbial diseases in animals. In some cases, this can be the result of faster dissemination of pathogens in specific climate conditions; such an example is the increased airborne transfer of respiratory pathogens with the wind [1]; other relevant references have referred to the potential for infection of animals by means of sharing contaminated water during hot periods [2], as well as the increased pathogen spread among animals due to congregation at watering points during the dry season [3]. In other instances, climate conditions may affect the ability of animals to respond to microbial infections effectively; such an example refers to the depletion of humoral and cellular components of immune reaction during low temperatures [4].

Vaccines provide significant protection against the respective infections, and long-term vaccination plans are important tools for achieving healthy individuals [5]. At the farm level, vaccinations have clear beneficial effects on the health and production of animals and are important constituents and determinants of the health management programs applied therein [6].

There is little information in the international literature regarding possible associations between climate factors and vaccinations in small ruminant farms. A search of the relevant literature was performed on the platform Web of Science; the search terms ‘[vaccin*] AND [climat* OR weather] AND [sheep OR goat*]’ were used. The initial results returned 98 records. Irrelevant records (e.g., referring to other animal species) were then excluded, and the remaining articles were individually assessed. This revealed that only nine articles precisely and fully covered the intended search. Among these articles, associations between climate factors and vaccination have been mostly studied for bluetongue; this is reasonable because climate factors play a clear role in the activity of the vectors for this *Orbivirus* pathogen [7,8,9]. It is also noteworthy that of these articles, six (67%) were published during the last five years, i.e., as concerns increase regarding climate change and its potential effects on farming, whilst, at the same time, technologies for improving animal health extend.

One can hypothesise that vaccine administration may, at least to some extent, be linked to climate conditions in farms. This may be particularly true for sheep and goat farming, as these animals are increasingly exposed to climate conditions due to their grazing habits. In this context, Thirunavukkarasu et al. [10] reported that in goat farms in India, vaccinations against various infections were practiced more frequently in farms where climatic conditions posed a lower risk for respective infections: 58% of farms versus 36% in farms with a higher risk of infections due to climatic factors. It is noted that, for the vaccination of people, prediction models for the optimum season for vaccination have been devised for some pathogens [11].

In Greece, sheep and goat farming is the most important livestock sector, generating 18% of the total primary sector income and 0.8% of the country’s total gross domestic product [12]. In a previous study, we described the patterns of vaccinations in sheep and goat farms in the country, and we presented the management- and human resources-related factors associated with the use of specific vaccines [13]. The results of that study revealed significant associations between the administration of various optional vaccines with 11 variables related to health management practices and 4 variables related to the demographic characteristics of farmers; among these, the collaboration with a veterinarian, the daily number of milking sessions and the period spent daily by the farmer at the farm premises were each associated with the administration of vaccines against three infections [13]. However, the potential effects of climate factors in the administration of vaccines have not been investigated and assessed.

The objectives of the present study were (a) to evaluate the importance of climate-related variables in the vaccination patterns applied in sheep and goat farms in Greece and (b) to assess potential interactions between these factors and previously established important health management- and human resources-related factors applied in the farms.

## 2. Materials and Methods

### 2.1. Data Collection and Management

**Figure 1:**
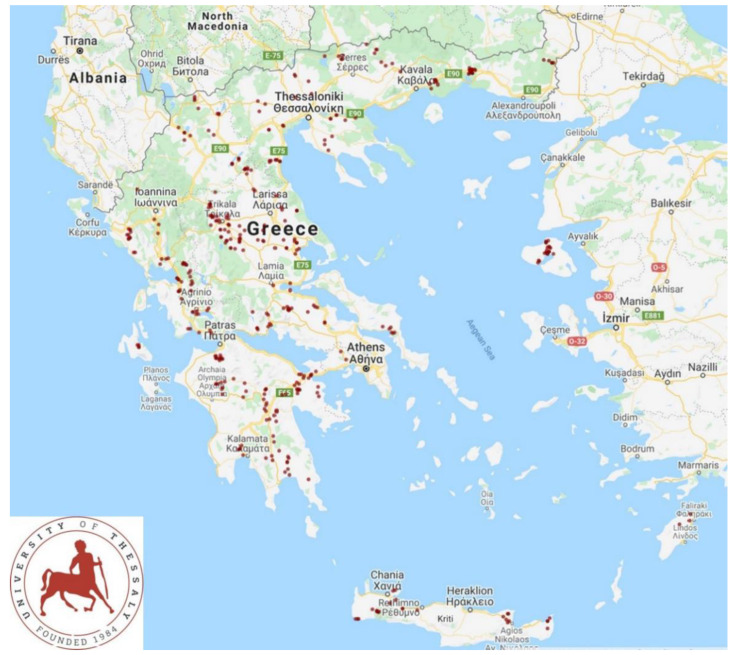
Map showing the locations of the 325 sheep flocks and 119 goat herds throughout Greece, which were visited to collect data and information on vaccination schedules and procedures.

The present work has been based on data obtained during an extensive cross-sectional study in sheep and goat farms, which was previously performed in small ruminant farms based throughout Greece [13] performed in 2020. In total, 444 farms (325 sheep flocks and 119 goat herds) were enrolled in the study; these were located in all 13 administrative regions of the country (Figure 1).

During the visit, information was gathered from the farmers by using a structured questionnaire [14] with regard to the patterns and applications of vaccinations performed against eight (8) infections of sheep and goats (specifically: chlamydial abortion, clostridial infections, contagious agalactia, contagious ecthyma, foot-rot, paratuberculosis, pneumonia and staphylococcal mastitis); vaccinations against all above infections were optional. Vaccination patterns evaluated referred to the reproductive cycle (i.e., mating to the cessation of milking) preceding the visit to the farm. Then, the total number of vaccinations performed on a farm was calculated.

Data on farm location were collected by means of portable Global Positioning System Garmin units. By using these, we resolved the geo-references to the specific farm level.

Climatic variables were obtained from ‘The POWER (Prediction of Worldwide Energy Resources) Project’ (NASA Langley Research Center (LaRC), Hampton, VA, USA), which provides meteorological datasets from NASA research for the support of agricultural needs. The following settings were used for obtaining the data: user community, ‘agroclimatology’; time extent, ‘start date 2010–end date 2019′; latitude/longitude, ‘geo-references of each farm’; temporal average, ‘monthly and annual’; and output file format, ‘ASCII’ [15]. Based on these settings, we obtained data for the parameters as follows: the temperature at 2 m (T2M), the temperature of Earth skin (TS), the temperature range at 2 m (T2Ran), the minimum temperature at 2 m (T2Min), the maximum temperature at 2 m (T2Max), precipitation (PREC), the relative humidity at 2 m (RH2m) and the wind speed at 10 m (WS10m).

For the evaluations, the annual averages provided by the above platform for every year from 2010 to 2019 (*n* = 10) were taken into account. Two sets of climatic variables were produced: one with results of ten years prior to the visit (2010 to 2019) and one with two years prior to the visit (2018 and 2019).

### 2.2. Data Analysis

Data were entered into Microsoft Excel and analysed using SPSS v. 26 (IBM Analytics, Armonk, NY, USA). A basic descriptive analysis was performed.

Initially, an analysis of variance was used to evaluate differences in climatic variables between (a) sheep flocks and goat herds, (b) farms with intensive or semi-intensive management and farms with extensive or semi-extensive management and (c) the two datasets (10-year and 2-year) used in the analysis.

The following nine outcomes were considered: ‘vaccination against chlamydial abortion’, ‘vaccination against clostridial infections’, ‘vaccination against contagious agalactia’, ‘vaccination against contagious ecthyma’, ‘vaccination against foot-rot’, ‘vaccination against paratuberculosis’, ‘vaccination against bacterial pneumonia’, ‘vaccination against staphylococcal mastitis’ and ‘total number of optional vaccines administered’. Univariable analyses were initially performed by applying analysis of variance and with simple logistic regression, separately for sheep flocks and for goat herds, as well as separately for farms with intensive or semi-intensive management and for farms with semi-extensive or extensive management (classified according to the criteria of the European Food Safety Authority [16]).

Subsequently, again separately for each outcome, a multivariable model was constructed. Climatic variables, in which *p* < 0.2 was found in the initial univariable analyses, were offered and included in this model. Progressively, variables were removed from the model by using backward elimination. The likelihood ratio test was performed to assess the *p*-value of each variable; among variables seen with *p* > 0.2, the variable with the largest *p*-value was taken out of the model. The procedure was repeated until no variable could be removed, i.e., with *p* > 0.2. That way, in total, 62 multivariable models were created and evaluated. The details of the variables within each of the above 62 multivariable models are shown in Table S1.

After establishing the significant climatic variables for each of the above nine outcomes ((a) separately for sheep flocks and for goat herds and (b) separately for farms with intensive or semi-intensive management and for farms with semi-extensive or extensive management), new multivariable analyses were performed for each outcome, which included (a) the climatic variables found to be significant for each outcome, as described above (*p* < 0.05), and (b) management-related or human resources-related parameters previously found to be significant for each of the above outcomes, respectively [13]. The same procedure as above, i.e., with progressive removal of variables with *p* > 0.2, was followed. That way, in total, 67 multivariable models were created and evaluated. The details of the variables within each of the above 67 multivariable models are shown in Table S2.

In all analyses, statistical significance was defined at *p* < 0.05.

## 3. Results

### 3.1. Climatic Conditions in the Farms

The climatic variables in the farms in the study are presented in Table 1. For six variables (the temperature at 2 m, the temperature of Earth skin, the temperature range at 2 m, the maximum temperature at 2 m, precipitation and relative humidity at 2 m), there were significant differences between the two datasets employed in the analysis. Specifically, in the 2-year dataset, the temperature of Earth skin and temperature at 2 m were higher, the temperature range at 2 m and maximum temperature at 2 m were comparatively lower and the precipitation and relative humidity at 2 m were also higher (Figure S1).

No significant differences in climatic variables were noted between sheep flocks and goat herds for both datasets. In contrast, significant differences in the variables were seen between farms under intensive or semi-intensive management and farms under semi-extensive and extensive management for both datasets.

### 3.2. Associations of Climatic Variables with Vaccinations

The detailed results of the univariable analyses for associations of the climatic variables assessed with vaccinations against the eight infections are presented in Tables S3–S10. For each outcome, separate findings are presented for sheep flocks/goat herds and for farms with intensive or semi-intensive management/farms with semi-extensive or extensive management. The detailed results of the subsequent multivariable analyses for associations of the climatic variables assessed with vaccinations against the eight infections are presented in Tables S11–S18. Again, separate findings are presented for sheep flocks/goat herds and for farms with intensive or semi-intensive management/farms with semi-extensive or extensive management.

Climatic variables had a higher association with vaccinations against infections in sheep flocks (26 associations) than in goat herds (9 associations) (*p* = 0.002) and in farms with semi-extensive or extensive management (32 associations) than in farms with intensive or semi-intensive management (8 associations) (*p* < 0.0001). There were no differences between the various climatic variables evaluated in their associations with vaccinations (*p* > 0.15). Moreover, there was no difference between the 10-year and the 2-year data in their association with vaccinations (*p* > 0.25). The cumulative findings of the multivariable analyses are presented in Table 2.

The detailed results of the univariable and multivariable analyses for associations with the total number of optional vaccinations are presented in Tables S19 and S20. For each outcome, overall findings are presented (Figure S2), as well as findings separately for sheep flocks/goat herds and for farms with intensive or semi-intensive management/farms with semi-extensive or extensive management (Tables S19 and S20).

### 3.3. Significant Predictors for Vaccinations

The detailed results of the multivariable analyses for associations of climatic parameters, management-related parameters or human resources-related parameters with vaccinations against the eight infections are presented in Tables S21–S28. The findings are presented separately for sheep flocks and goat herds and separately for farms with intensive or semi-intensive management and farms with semi-extensive or extensive management. The cumulative findings of these multivariable analyses are presented in Table S29.

The detailed results of the multivariable analyses for associations of climatic parameters, management-related parameters or human resources-related parameters with the total number of optional vaccinations are presented in Table S30.

**Figure 2:**
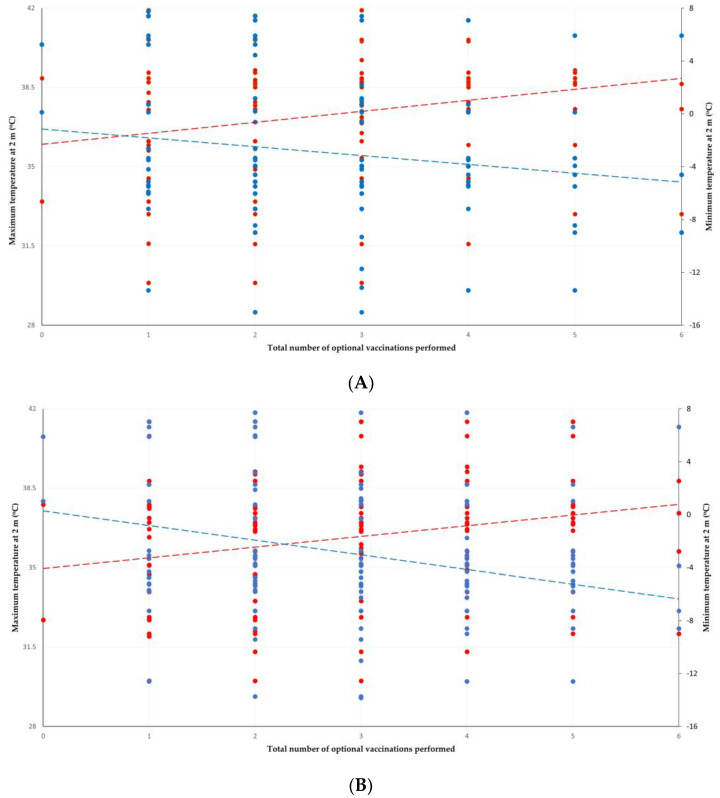
Scatter plots of the total number of optional vaccinations versus the maximum temperature at 2 m (red dots) and the minimum temperature at 2 m (blue dots) in small ruminant farms in Greece in which semi-extensive or extensive management is applied (dotted lines are respective trendlines): (**A**) the 10-year dataset with the most significant variable at the maximum temperature at 2 m (*p* = 0.0004), and (**B**) the 2-year dataset with the most significant variable at the minimum temperature at 2 m (*p* = 0.0002) (Table S29).

Climatic variables were found to be significant predictors in vaccination against all eight infections studied. Climatic variables had a higher association with vaccinations against infections in sheep flocks (22 associations) than in goat herds (5 associations) (*p* = 0.0005) and in farms with semi-extensive or extensive management (25 associations) than in farms with intensive or semi-intensive management (6 associations) (*p* = 0.0003). For all eight infections, there were changes in the climatic variables found to be significant predictors from the 10-year dataset to the 2-year dataset. For the total number of optional vaccinations performed, there were also changes in the climatic variables found to be significant predictors from the 10-year dataset to the 2-year dataset (Figure 2).

In 26 analyses (38.8%), the climatic variables overshadowed the management- and human resources-related variables assessed as significant predictors for vaccination (Table 1). In most cases, these referred to sheep flocks (9 analyses) and farms with semi-extensive or extensive management (8 analyses). Climatic variables identified as predictors are summarised in Table 3.

## 4. Discussion

### 4.1. Preamble

The present work combines the findings of previous studies performed in our group, draws information provided by previous works [13,15], capitalises on knowledge achieved through the previous studies and provides a fusion of previous data in a meta-analysis approach. To the best of our knowledge, this is the first attempt internationally to associate vaccination patterns in small ruminants with climatological conditions.

The results indicate that climatic factors can play a role in decisions for the vaccination programs applied in small ruminant farms. The eight infections that were assessed during the study are all significant problems, leading to suboptimal health and reduced production of affected sheep and goats. None of these infections is legally regulated and monitored, which would have led to compulsory vaccinations; hence, farmers and veterinarians are responsible for the decision to vaccinate the animals against these. In a previous publication, the potential effects of management-related and human resources-related factors have been outlined [13]. The results of that study revealed significant associations between the administration of various optional vaccines with 11 variables related to health management practices and 4 variables related to the demographic characteristics of farmers; among these, the collaboration with a veterinarian, the daily number of milking sessions and the period spent daily by the farmer at the farm premises were each associated with the administration of vaccines against three infections [13]. In the present analysis, we took a different approach and assessed the possible effects of climatic variables.

Reasons that may influence decisions to vaccinate animals based on climatic factors can include the effects of climatic factors on the development and the course of an infection, due, for example, to affecting the circulation of pathogens [17]. For example, the role of climatic factors in the development of pneumonia in sheep has been presented in detail by Lacasta et al. [18]. More recently, Vasileiou et al. [19] associated subclinical mastitis in sheep with temperature prior to the sampling date. Therefore, there is a background for associations of vaccination programs for sheep and goats with the climate conditions prevailing in the farms. Farmers may base their decisions on vaccinations in accordance with climate and weather patterns, i.e., on real threats, as well as their perceptions of potential disease risks during a forthcoming time period.

Indeed, ‘weather lore’ (i.e., the body of informal folklore relating to predicting the weather [20]) may also be responsible for decisions to perform vaccination of animals. In the Greek countryside, this weather lore accumulates in the ‘*merominia*’ cliché, which is used to ‘predict’ the weather in the forthcoming winter [21,22]. Given that these predictions are based on the August weather [21,22] and that vaccinations in small ruminant farms are more often carried out from September to October (during the dry period of the ewes), it is understandable the potential influence of these ‘predictions’ in the vaccinations schedules of the farms. To note that similar empirical meteorological approaches can be found in the culture of other countries, e.g., the ‘*cabañuelas*’ system in Spain and Mexico [23].

### 4.2. Relevance of Findings

Among sheep flocks, there was a higher proportion of farms with animals of imported breeds (e.g., Lacaune, Assaf, etc.: 42.8% of all flocks) or improved-high productivity Greek breeds (Chios, Friesarta: 22.8%) than of farms with indigenous breeds (e.g., Boutsko, Sfakia, etc.: 4.3%), whilst contrasting findings were seen among goat herds (Murciano-Granadina, Saanen, etc.: 22.6% of all herds; Skopelos: 2.5%; and indigenous *Capra prisca*: 25.6%) [24]. In general, indigenous breeds of sheep and goats are considered to be less susceptible to disease threats than many mainstream, high-production breeds [25,26], and relevant findings have also been reported regarding Greek breeds [27,28]. Moreover, indigenous breeds of sheep and goats are considered to be more tolerant to adverse weather conditions; previous studies have reported genetic differences in adaptation to temperature stress and, thus, resilience to extreme temperatures [29,30], which are mediated through a complex network of genes [31,32]. In general, local breeds of small ruminants (or their crosses) are believed to have better tolerance to local climate conditions than imported breeds, and relevant findings have been published from work performed in various areas internationally, e.g., Africa [33], Brazil [34] and India [35]. Therefore, climate factors can exert a more pronounced effect on animals more susceptible to various disease threats, which would thus be a greater need for protective measures. The above hypothesis is aligned with the findings of a higher association of climate variables with vaccinations against the various infections in sheep flocks.

The frequent associations of climatic variables with vaccinations in farms with semi-extensive or extensive management can be the consequence of the more pronounced effects of environmental factors in those farms. However, there may be another hypothesis as significantly more farmers practicing semi-extensive or extensive management follow the family tradition in farming work than farmers practicing intensive or semi-intensive management (92% versus 82%, respectively) [36]; it may also be possible that they take into account the weather lore more often and more seriously, which thus influences vaccination plans. This hypothesis refers to an association of vaccinations as a means to prevent diseases with perceived adverse weather conditions facilitating such outbreaks, as there is an established relationship between climate and disease development.

The changes in climatic factors are in accordance with the time period considered, associated with vaccination schedules in the farms of the study and reflect the progressive changes in the climate variables in the agricultural environment of these farms, as shown in a previous paper [15]. These changes may modify disease threats for animals, as also shown in that work [15], and, consequently, the needs and perceptions for formulating vaccination programs. Moreover, they may influence decision-making as they continuously evolve. During the summer 2014 epidemic of bluetongue in Greece, the authorities did not allow vaccinations of animals, expecting that, during the incoming winter, there would have been decreased flying activity of vectors of the pathogen and, consequently, dissemination of the infection; however, due to the high temperatures that prevailed in the country that year, cases of the infection were diagnosed in December 2014 and January 2015 (which had not been forecast and expected), which led to an overturn of previous decisions and finally to the emergency licencing of vaccines for administration to sheep and goats. That measure contributed significantly to limiting the infection during the second year of the incursion [37,38].

The potential effect of climatic factors may be direct, i.e., as climatic factors influence infections, the need for vaccination develops. It is also recognised that the effect may possibly be presumptive, i.e., as farmers forecast (correctly or arbitrarily) forthcoming weather conditions that can favour diseases (based on their empirical knowledge), they vaccinate their animals in order to decrease the perceived risk of disease development. The finding of more significant associations of vaccinations with climatic data in farms managed under the semi-extensive or extensive system, i.e., where animals are more exposed to weather conditions, lends support to the above ideas.

## 5. Conclusions

The results indicated that, in some cases, climate factors overshadowed factors traditionally considered for the formulation of vaccination programs. This will be particularly useful within the context of precision sheep and goat farming. Indeed, climate variables are particularly important in livestock farming on extensive systems [39], where such approaches can provide higher efficiency and concurrent sustainability in the farms by improving the welfare of animals in the farms [40].

Nevertheless, it is pointed out that the associations recorded in the present study may not always be the direct effect of climatic factors on disease outbreaks and may not reflect an association of the need to protect against infection outbreaks as climatic conditions change. These may also be the results of perceptions of farmers, as well as the consequences of an indirect influence of climatic factors on disease patterns (e.g., through the effects on tolerance and susceptibility of the various animal breeds to varying weather conditions).

Future studies can be focused on formulating vaccination programs in accordance with climate factors and also on setting the optimum season(s) for vaccination of animals based on the circulation of the pathogens, the risks for the development of diseases and the stage within the annual production cycle of the animals. Potentially, an algorithm could be designed to take into account climate factors for the formulation of vaccination programs in small ruminant farms. This can take into account variables found in this study to be important for the vaccination programs, which will put into clinical practice the context of precision livestock farming.

## Figures and Tables

**Table 1:** Mean (±standard error) for climatic variables that prevailed in the farms during the 10 and 2 years prior to the study.

Climatic Variable	10-Year Average				2-Year Average			
	Sheep Flocks	Goat Herds	Farms with I and s-I ^2^	Farms with s-E and E ^2^	Sheep Flocks	Goat Herds	Farms with I and s-I ^1^	Farms with s-E and E
Temperature at 2 m ^1^	15.8 ± 0.1 ^c^	15.7 ± 0.2	15.3 ± 0.1 ^a,e^	16.2 ± 0.1 ^a,f^	16.1 ± 0.1 ^c^	16.0 ± 0.2	15.7 ± 0.1 ^b,e^	16.5 ± 0.1 ^b,f^
Temperature of Earth Skin ^1^	16.2 ± 0.1 ^c^	16.1 ± 0.2	15.6 ± 0.1 ^a,e^	16.8 ± 0.2 ^a^	16.5 ± 0.1 ^c^	16.4 ± 0.2	15.9 ± 0.1 ^b,e^	17.1 ± 0.2 ^b^
Temperature Range at 2 m ^1^	42.2 ± 0.3 ^c^	42.0 ± 0.6 ^d^	44.3 ± 0.3 ^a,e^	40.0 ± 0.5 ^a,f^	40.6 ± 0.3 ^c^	40.4 ± 0.6 ^d^	42.8 ± 0.3 ^b,e^	38.3 ± 0.5 ^b,f^
Minimum Temperature at 2 m ^1^	−4.1 ± 0.3	−4.3 ± 0.5	−5.5 ± 0.3 ^a^	−2.8 ± 0.3 ^a^	−3.7 ± 0.3	−4.0 ± 0.6	−5.3 ± 0.3 ^b^	−2.3 ± 0.4 ^b^
Maximum Temperature at 2 m ^1^	37.7 ± 0.2 ^c^	37.7 ± 0.2 ^d^	38.2 ± 0.3 ^a,e^	37.2 ± 0.2 ^a,f^	36.5 ± 0.2 ^c^	36.5 ± 0.2 ^d^	37.0 ± 0.3 ^b,e^	36.1 ± 0.2 ^b,f^
Precipitation ^1^	1.7 ± 0.1 ^c^	1.7 ± 0.1 ^d^	1.7 ± 0.1 ^e^	1.7 ± 0.1 ^f^	1.9 ± 0.1 ^c^	1.9 ± 0.1 ^d^	1.9 ± 0.1 ^b,e^	2.0 ± 0.1 ^b,f^
Relative Humidity at 2 m ^1^	68.6 ± 0.1 ^c^	68.9 ± 0.2 ^d^	68.1 ± 0.1 ^a,e^	69.3 ± 0.1 ^a,f^	69.2 ± 0.1 ^c^	69.4 ± 0.2 ^d^	68.6 ± 0.1 ^b,e^	69.9 ± 0.1 ^b,f^
Wind Speed at 10 m ^1^	2.4 ± 0.1	2.4 ± 0.1	2.1 ± 0.1 ^a^	2.7 ± 0.1 ^a^	2.4 ± 0.1	2.4 ± 0.1	2.1 ± 0.1 ^b^	2.7 ± 0.1 ^b^

^1^ temperature variables: expressed in °C, precipitation expressed in mm, relative humidity expressed as % and wind speed expressed in m s^−1^. ^2^ I: intensive, E: extensive and s: semi. ^a,b,c,d,e,f^: figures with the same superscript within the same row differ significantly (*p* < 0.05).

**Table 2:** Cumulative findings of the multivariable analysis of climate conditions for associations with optional vaccination against eight infections ^1^ in 444 small ruminant farms in Greece.

Climatic Variable	10-Year Average				2-Year Average			
	Sheep Flocks	Goat Herds	Farms with I and s-I ^2^	Farms with s-E and E ^2^	Sheep Flocks	Goat Herds	Farms with I and s-I ^1^	Farms with s-E and E
Temperature at 2 m	ci			ca, ci, cag	ci, pa		ce	cag, sm
Temperature of Earth Skin	ci		pn	ci, sm	ci			sm
Temperature Range at 2 m	ce	pn		cag, fr, ce	ce			cag, ce
Minimum Temperature at 2 m	ce	pn	pa	fr	ce			ce
Maximum Temperature at 2 m	ce, pn			cag, fr, ce, pa	ce, pn		pn	cag, cen
Relative Humidity at 2 m	ci, cag, pn	pn		ca, ci, pn	ci, cag, pn	pn	fr, pn	pn
Precipitation	ci	ca, cag				cag		ca, sm
Wind speed at 10 m	ca, cag, sm		cag	ca, ci, sm	ca, cag, sm	ca, sm	cag	ca, sm

^1^ ca: chlamydial abortion, ci: clostridial infections, cag: contagious agalactia, ce: contagious ecthyma, fr: foot-rot, pa: paratuberculosis, pn: pneumonia and sm: staphylococcal mastitis. ^2^ I: intensive, E: extensive and s: semi.

**Table 3:** Climatic parameters that were found to be associated with optional vaccination against eight infections in 444 small ruminant farms in Greece.

Infection	10-Year Average	2-Year Average
Chlamydial Abortion	T2M (s-E and E), RH2m (s-E and E), WS10m (s-E and E) ^1^	PREC (s-E and E), WS10m (S)
Clostridial Infections	TS (S, s-E and E), T2M (S, s-E and E), RH2m (S), PREC (S), WS10m (s-E and E)	TS (S), T2M (S), RH2m (S)
Contagious Agalactia	T2M (s-E and E), T2Max (s-E and E), T2Ran (s-E and E), S10m (S, G, I and s-I)	T2M (s-E and E), T2Max (s-E and E), T2Ran (s-E and E), RH2m (S), WS10m (S, G, I and s-I)
Contagious Ecthyma	T2Max (S), T2Min (S), T2Ran (S)	T2M (S), T2Max (S, s-E and E), T2Min (S, s-E and E), T2Ran (S, s-E and E)
Foot-rot	T2Max (s-E and E), T2Min (s-E and E), T2Ran (s-E and E)	RH2m (I and s-I)
Paratuberculosis	T2Min (I and s-I)	-
Pneumonia	T2Max (S), T2Min (G), T2Ran (G), RH2m (S, G, I and s-I, s-E and E)	T2Max (S), RH2m (S, I and s-I, s-E and E)
Staphylococcal Mastitis	-	TS (s-E and E), T2M (s-E and E), PREC (s-E and E), WS10m (s-E and E)

^1^ TS: the temperature of Earth skin, T2M: the temperature at 2 m, T2Max: the maximum temperature at 2 m, T2Min: the minimum temperature at 2 m, T2Ran: the temperature range at 2 m, RH2m: the relative humidity at 2 m, PREC: precipitation, WS10m: the wind speed at 10 m—S: sheep, G: goat, I: intensive, E: extensive and s: semi.

## Data Availability

Most data presented in this study are in the Supplementary Materials.

## Supplementary Materials

The following supporting information can be downloaded at: https://www.mdpi.com/article/10.3390/vaccines11040797/s1, Table S1: Details of multivariable models employed for the evaluation of potential associations with optional vaccinations in 444 small ruminant farms in Greece; Table S2: Details of multivariable models employed in the comparative evaluation for potential comparative associations of management-related, human resources-related and climate-related variables with optional vaccinations in 444 small ruminant farms in Greece; Figure S1: Visual presentation of differences in the climatic variables assessed for associations with vaccination against infections in 444 small ruminant farms in Greece, with comparisons made between the 10-year (2010–2019) and the 2-year (2018–2019) datasets; Table S3: Results of univariable analysis of climate conditions for associations with optional vaccination against chlamydial abortion in 444 small ruminant farms in Greece; Table S4: Results of univariable analysis of climate conditions for associations with optional vaccination against clostridial infections in 444 small ruminant farms in Greece; Table S5: Results of univariable analysis of climate conditions for associations with optional vaccination against contagious agalactia in 444 small ruminant farms in Greece; Table S6: Results of univariable analysis of climate conditions for associations with optional vaccination against contagious ecthyma in 444 small ruminant farms in Greece; Table S7: Results of univariable analysis of climate conditions for associations with optional vaccination against foot-rot in 444 small ruminant farms in Greece; Table S8: Results of univariable analysis of climate conditions for associations with optional vaccination against paratuberculosis in 444 small ruminant farms in Greece; Table S9: Results of univariable analysis of climate conditions for associations with optional vaccination against pneumonia in 444 small ruminant farms in Greece; Table S10: Results of univariable analysis of climate conditions for associations with optional vaccination against staphylococcal mastitis in 444 small ruminant farms in Greece; Table S11: Results of multivariable analysis of climate conditions for associations with optional vaccination against chlamydial abortion in 444 small ruminant farms in Greece; Table S12: Results of multivariable analysis of climate conditions for associations with optional vaccination against clostridial infections in 444 small ruminant farms in Greece; Table S13: Results of multivariable analysis of climate conditions for associations with optional vaccination against contagious agalactia in 444 small ruminant farms in Greece; Table S14: Results of multivariable analysis of climate conditions for associations with optional vaccination against contagious ecthyma in 444 small ruminant farms in Greece; Table S15: Results of multivariable analysis of climate conditions for associations with optional vaccination against foot-rot in 444 small ruminant farms in Greece; Table S16: Results of multivariable analysis of climate conditions for associations with optional vaccination against paratuberculosis in 444 small ruminant farms in Greece; Table S17: Results of multivariable analysis of climate conditions for associations with optional vaccination against pneumonia in 444 small ruminant farms in Greece; Table S18: Results of multivariable analysis of climate conditions for associations with optional vaccination against staphylococcal mastitis in 444 small ruminant farms in Greece; Table S19: Results of univariable analysis of climate conditions for associations with the total number of optional vaccinations in 444 small ruminant farms in Greece; Table S20: Results of multivariable analysis of climate conditions for associations with the total number of optional vaccinations in 444 small ruminant farms in Greece; Figure S2: Scatter plots of the total number of optional vaccinations versus the maximum temperature at 2 m and the minimum temperature at 2 m in 444 small ruminant farms in Greece; Table S21: Results of multivariable analysis for significant climatic, management-related or human resources-related parameters associated with optional vaccination against chlamydial abortion in 444 small ruminant farms in Greece; Table S22: Results of multivariable analysis for significant climatic, management-related or human resources-related parameters associated with optional vaccination against clostridial infections in 444 small ruminant farms in Greece; Table S23: Results of multivariable analysis for significant climatic, management-related or human resources-related parameters associated with optional vaccination against contagious agalactia in 444 small ruminant farms in Greece; Table S24: Results of multivariable analysis for significant climatic, management-related or human resources-related parameters associated with optional vaccination against contagious ecthyma in 444 small ruminant farms in Greece; Table S25: Results of multivariable analysis for significant climatic, management-related or human resources-related parameters associated with optional vaccination against foot-rot in 444 small ruminant farms in Greece; Table S26: Results of multivariable analysis for significant climatic, management-related or human resources-related parameters associated with optional vaccination against paratuberculosis in 444 small ruminant farms in Greece; Table S27: Results of multivariable analysis for significant climatic, management-related or human resources-related parameters associated with optional vaccination against pneumonia in 444 small ruminant farms in Greece; Table S28: Results of multivariable analysis for significant climatic, management-related or human resources-related parameters associated with optional vaccination against staphylococcal mastitis in 444 small ruminant farms in Greece; Table S29: Summary of multivariable analyses for outcomes regarding optional vaccinations in small ruminant farms in Greece; Table S30: Results of multivariable analysis for significant climatic, management-related or human resources-related parameters associated with the total number of optional vaccinations in 444 small ruminant farms in Greece.
