# Supplementary material for: Relationship between Vaccine Application and Climate Factors in Sheep and Goat Farms in Greece
Source: Vaccines (Basel). 2023 Apr 4;11(4):797. doi: 10.3390/vaccines11040797 (PMC10142817; doi:10.3390/vaccines11040797)
Supplement: Supplementary file 1 [file vaccines-11-00797-s001.zip › vaccines-2270076-supplementary.pdf]

Relationship between Vaccine Application and Climate Factors in Sheep and Goat Farms in Greece

Eleni I. Katsarou and George C. Fthenakis

Table S1. Details of multivariable models (n = 62) employed for the evaluation of potential associations with optional vaccinations in 444 small ruminant farms in Greece.

Outcome	Variables	
	**offered to the multi-variable models (n)**	**required in the final models**
Vaccination against chlamydial abortion – sheep – 10-year average	7	(a) minimum temperature at 2 m, (b) relative humidity at 2 m, (c) wind speed at 10 m
Vaccination against chlamydial abortion – sheep – 2-year average	7	(a) minimum temperature at 2 m, (b) relative humidity at 2 m, (c) wind speed at 10 m
Vaccination against chlamydial abortion – goats – 10-year average	7	(a) precipitation
Vaccination against chlamydial abortion – goats – 2-year average	7	(a) wind speed at 10 m
Vaccination against chlamydial abortion – I & s-I [1] – 10-year average	0	n/a
Vaccination against chlamydial abortion – I & s-I – 2-year average	0	n/a
Vaccination against chlamydial abortion – s-E & E – 10-year average	8	(a) temperature at 2 m, (b) relative humidity at 2 m, (c) wind speed at 10 m
Vaccination against chlamydial abortion – s-E & E – 2-year average	7	(a) precipitation, (b) wind speed at 10 m
Vaccination against clostridial infections – sheep – 10-year average	8	(a) temperature of Earth skin, (b) temperature at 2 m, (c) relative humidity at 2 m, (d) precipitation
Vaccination against clostridial infections – sheep – 2-year average	8	(a) temperature of Earth skin, (b) temperature at 2 m, (c) relative humidity at 2 m, (d) precipitation
Vaccination against clostridial infections – goats – 10-year average	1	(a) relative humidity at 2 m
Vaccination against clostridial infections – goats – 2-year average	2	(a) relative humidity at 2 m
Vaccination against clostridial infections – I & s-I – 10-year average	0	n/a
Vaccination against clostridial infections – I & s-I – 2-year average	0	n/a

Vaccination against clostridial infections – s-E & E – 10-year average	8	(a) temperature of Earth skin, (b) temperature at 2 m, (c) wind speed at 10 m
Vaccination against clostridial infections – s-E & E – 2-year average	7	(a) precipitation
Vaccination against contagious agalactia – sheep – 10-year average	8	(a) relative humidity at 2 m, (b) wind speed at 10 m
Vaccination against contagious agalactia – sheep – 2-year average	7	(a) relative humidity at 2 m, (b) wind speed at 10 m
Vaccination against contagious agalactia – goats – 10-year average	7	(a) precipitation, (b) wind speed at 10 m
Vaccination against contagious agalactia – goats – 2-year average	7	(a) maximum temperature at 2 m, (b) wind speed at 10 m
Vaccination against contagious agalactia – I & s-I – 10-year average	7	(a) wind speed at 10 m
Vaccination against contagious agalactia – I & s-I – 2-year average	6	(a) temperature at 2 m, (b) minimum temperature at 2 m, (c) relative humidity at 2 m, (d) wind speed at 10 m
Vaccination against contagious agalactia – s-E & E – 10-year average	8	(a) temperature at 2 m, (b) maximum temperature at 2 m, (c) temperature range at 2 m
Vaccination against contagious agalactia – s-E & E – 2-year average	7	(a) temperature at 2 m, (b) minimum temperature at 2 m, (c) temperature range at 2 m
Vaccination against contagious ecthyma – sheep – 10-year average	6	(a) maximum temperature at 2 m, (b) minimum temperature at 2 m, (c) temperature range at 2 m, (d) relative humidity at 2 m
Vaccination against contagious ecthyma – sheep – 2-year average	5	(a) temperature of Earth skin, (b) temperature at 2 m, (c) maximum temperature at 2 m, (d) minimum temperature at 2 m,
Vaccination against contagious ecthyma – goats – 10-year average	0	n/a
Vaccination against contagious ecthyma – goats – 2-year average	5	(a) wind speed at 10 m
Vaccination against contagious ecthyma – I & s-I – 10-year average	6	(a) temperature at 2 m
Vaccination against contagious ecthyma – I & s-I – 2-year average	5	(a) temperature of Earth skin, (b) temperature at 2 m
Vaccination against contagious ecthyma – s-E & E – 10-year average	6	(a) temperature of Earth skin, (b) maximum temperature at 2 m, (c) temperature range at 2 m
Vaccination against contagious ecthyma – s-E & E – 2-year average	8	(a) temperature at 2 m, (b) maximum temperature at 2 m, (c) minimum temperature at 2 m, (d) temperature range at 2 m
Vaccination against foot-rot – sheep – 10-year average	0	n/a
Vaccination against foot-rot – sheep – 2-year average	1	--

Vaccination against foot-rot – goats – 10-year average	0	n/a
Vaccination against foot-rot – goats – 2-year average	0	n/a
Vaccination against foot-rot – I & s-I – 10-year average	0	n/a
Vaccination against foot-rot – I & s-I – 2-year average	1	(a) relative humidity at 2 m
Vaccination against foot-rot – s-E & E – 10-year average	8	(a) maximum temperature at 2 m, (b) minimum temperature at 2 m, (c) temperature range at 2 m
Vaccination against foot-rot – s-E & E – 2-year average	8	(a) maximum temperature at 2 m, (b) minimum temperature at 2 m, (c) temperature range at 2 m
Vaccination against paratuber-culosis – sheep – 10-year average	4	(a) temperature of Earth skin, (b) temperature at 2 m
Vaccination against paratuber-culosis – sheep – 2-year average	4	(a) temperature of Earth skin, (b) temperature at 2 m
Vaccination against paratuber-culosis – goats – 10-year average	1	(a) maximum temperature at 2 m
Vaccination against paratuber-culosis – goats – 2-year average	1	(a) maximum temperature at 2 m
Vaccination against paratuber-culosis – I & s-I – 10-year average	5	(α) minimum temperature at 2 m, (b) relative humidity at 2 m
Vaccination against paratuber-culosis – I & s-I – 2-year average	5	(a) temperature of Earth skin, (b) temperature at 2 m, (c) minimum temperature at 2 m
Vaccination against paratuber-culosis – s-E & E – 10-year average	2	(a) maximum temperature at 2 m
Vaccination against paratuber-culosis – s-E & E – 2-year average	2	(a) maximum temperature at 2 m
Vaccination against pneumonia – sheep – 10-year average	2	(a) maximum temperature at 2 m, (b) relative humidity at 2 m
Vaccination against pneumonia – sheep – 2-year average	2	(a) temperature range at 2 m, (b) relative humidity at 2 m
Vaccination against pneumonia – goats – 10-year average	5	(a) minimum temperature at 2 m, (b) temperature range at 2 m, (c) relative humidity at 2 m, (d) precipitation
Vaccination against pneumonia – goats – 2-year average	5	(a) minimum temperature at 2 m, (b) temperature range at 2 m, (c) relative humidity at 2 m
Vaccination against pneumonia – I & s-I – 10-year average	6	(a) temperature of Earth skin, (b) temperature at 2 m, (c) maximum temperature at 2 m
Vaccination against pneumonia – I & s-I – 2-year average	7	(a) temperature of Earth skin, (b) temperature at 2 m, (c) maximum temperature at 2 m, (d) relative humidity at 2 m, (e) wind speed at 10 m
Vaccination against pneumonia – s-E & E – 10-year average	1	(a) relative humidity at 2 m

Vaccination against pneumonia – s-E & E – 2-year average	2	(a) relative humidity at 2 m
Vaccination against staphylococcal mastitis – sheep – 10-year average	4	(a) wind speed at 10 m
Vaccination against staphylococcal mastitis – sheep – 2-year average	4	(a) temperature range at 2 m, (b) wind speed at 10 m
Vaccination against staphylococcal mastitis – goats – 10-year average	4	(a) wind speed at 10 m
Vaccination against staphylococcal mastitis – goats – 2-year average	4	(a) temperature at 2 m, (b) minimum temperature at 2 m, (c) wind speed at 10 m
Vaccination against staphylococcal mastitis – I & s-I – 10-year average	1	(a) minimum temperature at 2 m
Vaccination against staphylococcal mastitis – I & s-I – 2-year average	1	(a) precipitation
Vaccination against staphylococcal mastitis – s-E & E – 10-year average	5	(a) temperature of Earth skin, (b) temperature at 2 m, (c) precipitation, (d) wind speed at 10 m
Vaccination against staphylococcal mastitis – s-E & E – 2-year average	5	(a) temperature of Earth skin, (b) temperature at 2 m, (c) precipitation, (d) wind speed at 10 m
Total number of optional vaccines administered – sheep – 10-year average	7	(a) temperature of Earth skin (b) maximum temperature at 2 m, (c) minimum temperature at 2 m, (d) temperature range at 2 m
Total number of optional vaccines administered – sheep – 2-year average	7	(a) temperature of Earth skin (b) maximum temperature at 2 m, (c) minimum temperature at 2 m, (d) wind speed at 10 m
Total number of optional vaccines administered – goats – 10-year average	6	(a) temperature at 2 m, (b) maximum temperature at 2 m, (c) temperature range at 2 m
Total number of optional vaccines administered – goats – 2-year average	6	(a) temperature at 2 m, (b) maximum temperature at 2 m, (c) maximum temperature at 2 m (d) temperature range at 2 m
Total number of optional vaccines administered – I & s-I – 10-year average	2	--
Total number of optional vaccines administered – I & s-I – 2-year average	0	n/a
Total number of optional vaccines administered – s-E & E – 10-year average	7	(a) temperature at 2 m, (b) maximum temperature at 2 m (c) temperature range at 2 m, (d) precipitation
Total number of optional vaccines administered – s-E & E – 2-year average	7	(a) temperature at 2 m, (b) maximum temperature at 2 m (c) temperature range at 2 m, (d) precipitation

1 I: intensive, E: extensive, s: semi.

Table S2. Details of multivariable models (*n* = 67) employed in the comparative evaluation for potential comparative associations of management-related, human resources-related and climate-related variables with optional vaccinations in 444 small ruminant farms in Greece.

Outcome	Variables	
	offered to the multi-variable models (*n*)	required in the final models
Vaccination against chlamydial abortion – sheep – 10-year average	3	(a) Age of newborns when taken away from dam, (b) Daily number of milking sessions, (c) Wind speed at 10 m
Vaccination against chlamydial abortion – sheep – 2-year average	3	(a) Age of newborns when taken away from dam, (b) Daily number of milking sessions, (c) Wind speed at 10 m
Vaccination against chlamydial abortion – goats – 10-year average	6	(a) Age of newborns when taken away from dam, (b) Daily number of milking sessions, (c) Wind speed at 10 m
Vaccination against chlamydial abortion – goats – 2-year average	6	(a) Breed of animals in the farm, (b) Average age of culling female animals, (c) Age of newborns when taken away from dam, (d) Daily number of milking sessions, (e) Daily period spent by farmer at the farm
Vaccination against chlamydial abortion – I & s-I [1] – 10-year average	5	(a) Age of newborns when taken away from dam, (b) Daily number of milking sessions,
Vaccination against chlamydial abortion – I & s-I – 2-year average	5	(a) Age of newborns when taken away from dam, (b) Daily number of milking sessions,
Vaccination against chlamydial abortion – s-E & E – 10-year average	8	(a) Average age of culling female animals, (b) Age of newborns when taken away from dam, (c) Daily period spent by farmer at the farm, (d) Temperature at 2m, (e) Relative humidity at 2 m, (f) Wind speed at 10 m
Vaccination against chlamydial abortion – s-E & E – 2-year average	7	(a) Average age of culling female animals, (b) Age of newborns when taken away from dam, (c) Daily period spent by farmer at the farm, (d) Precipitation, (e) Wind speed at 10 m
Vaccination against clostridial infections – sheep – 10-year average	7	(a) Age of farmers, (b) Family tradition in farming, (c) temperature of Earth skin, (d) temperature at 2 m, (e) relative humidity at 2 m, (f) precipitation
Vaccination against clostridial infections – sheep – 2-year average	6	(a) Management system applied in the farm, (b) Age of farmers, (c) Family tradition in farming, (d) temperature of Earth skin, (e) temperature at 2 m, (f) relative humidity at 2 m
Vaccination against clostridial infections – goats – 10-year average	1	(a) Family tradition in farming
Vaccination against clostridial infections – goats – 2-year average	1	(a) Family tradition in farming
Vaccination against clostridial infections – I & s-I – 10-year average	2	(a) Family tradition in farming

Vaccination against clostridial infections – I & s-I – 2-year average	2	(a) Family tradition in farming
Vaccination against clostridial infections – s-E & E – 10-year average	5	(a) Age of farmers, (b) Family tradition in farming, (c) temperature of Earth skin, (d) temperature at 2 m, (e) wind speed at 10 m
Vaccination against clostridial infections – s-E & E – 2-year average	2	(a) Family tradition in farming
Vaccination against contagious agalactia – sheep – 10-year average	5	(a) Management system applied in the farm, (b) Collaboration with a veterinarian, (c) Duration of dry-period, (d) Relative humidity at 2 m, (e) Wind speed at 10 m
Vaccination against contagious agalactia – sheep – 2-year average	5	(a) Management system applied in the farm, (b) Collaboration with a veterinarian, (c) Duration of dry-period, (d) Relative humidity at 2 m, (e) Wind speed at 10 m
Vaccination against contagious agalactia – goats – 10-year average	3	(a) Collaboration with a veterinarian, (b) Use of laboratory diagnostic examinations in samples of milk, (c) Wind speed at 10 m
Vaccination against contagious agalactia – goats – 2-year average	4	(a) Collaboration with a veterinarian, (b) Use of laboratory diagnostic examinations in samples of milk, (c) Wind speed at 10 m
Vaccination against contagious agalactia – I & s-I – 10-year average	4	(a) Use of laboratory diagnostic examinations in samples of milk, (b) Duration of dry-period, (c) Wind speed at 10 m
Vaccination against contagious agalactia – I & s-I – 2-year average	4	(a) Use of laboratory diagnostic examinations in samples of milk, (b) Wind speed at 10 m
Vaccination against contagious agalactia – s-E & E – 10-year average	6	(a) Collaboration with a veterinarian, (b) Use of laboratory diagnostic examinations in samples of milk, (c) Temperature at 2 m, (d) Maximum temperature at 2m, (e) Temperature range at 2 m
Vaccination against contagious agalactia – s-E & E – 2-year average	6	(a) Collaboration with a veterinarian, (b) Use of laboratory diagnostic examinations in samples of milk, (c) Temperature at 2 m, (d) Maximum temperature at 2m, (e) Temperature range at 2 m
Vaccination against contagious ecthyma – sheep – 10-year average	5	(a) Farmer's general education, (b) maximum temperature at 2 m, (c) minimum temperature at 2 m, (d) temperature range at 2 m
Vaccination against contagious ecthyma – sheep – 2-year average	5	(a) Farmer's general education, (b) maximum temperature at 2 m, (c) minimum temperature at 2 m, (d) temperature range at 2 m
Vaccination against contagious ecthyma – goats – 10-year average	0	n/a
Vaccination against contagious ecthyma – goats – 2-year average	0	n/a

Vaccination against contagious ecthyma – I & s-I – 10-year average	2	(a) Farmer's general education
Vaccination against contagious ecthyma – I & s-I – 2-year average	3	(a) Farmer's general education, (b) temperature at 2 m
Vaccination against contagious ecthyma – s-E & E – 10-year average	4	(a) Average age of culling female animals, (b) Farmer's general education
Vaccination against contagious ecthyma – s-E & E – 2-year average	5	(a) Farmer's general education, (b) maximum temperature at 2 m, (c) minimum temperature at 2 m, (d) temperature range at 2 m
Vaccination against foot-rot – sheep – 10-year average	1	(a) Farmer's general education
Vaccination against foot-rot – sheep – 2-year average	1	(a) Farmer's general education
Vaccination against foot-rot – goats – 10-year average	0	n/a
Vaccination against foot-rot – goats – 2-year average	0	n/a
Vaccination against foot-rot – I & s-I – 10-year average	1	(a) Farmer's general education
Vaccination against foot-rot – I & s-I – 2-year average	2	(a) Farmer's general education, (b) Relative humidity at 2 m
Vaccination against foot-rot – s-E & E – 10-year average	4	(a) Farmer's general education, (b) maximum temperature at 2 m, (c) minimum temperature at 2 m, (d) temperature range at 2 m
Vaccination against foot-rot – s-E & E – 2-year average	1	(a) Farmer's general education
Vaccination against paratuber-culosis – sheep – 10-year average	0	n/a
Vaccination against paratuber-culosis – sheep – 2-year average	1	(a) temperature at 2 m
Vaccination against paratuber-culosis – goats – 10-year average	1	(a) No. of female animals in the farm
Vaccination against paratuber-culosis – goats – 2-year average	1	(a) No. of female animals in the farm
Vaccination against paratuber-culosis – I & s-I – 10-year average	2	(a) minimum temperature at 2 m
Vaccination against paratuber-culosis – I & s-I – 2-year average	1	--
Vaccination against paratuber-culosis – s-E & E – 10-year average	2	(a) maximum temperature at 2 m
Vaccination against paratuber-culosis – s-E & E – 2-year average	1	--

Vaccination against pneumonia – sheep – 10-year average	4	(a) Routine administration of antibiotics to newborns, (b) Period spent daily by farmer at the farm premises, (c) maximum temperature at 2 m, (d) relative humidity at 2 m
Vaccination against pneumonia – sheep – 2-year average	4	(a) Routine administration of antibiotics to newborns, (b) Period spent daily by farmer at the farm premises, (c) maximum temperature at 2 m, (d) relative humidity at 2 m
Vaccination against pneumonia – goats – 10-year average	3	(a) minimum temperature at 2 m, (b) temperature range at 2 m, (c) relative humidity at 2m
Vaccination against pneumonia – goats – 2-year average	1	(a) relative humidity at 2 m
Vaccination against pneumonia – I & s-I – 10-year average	3	(a) Period spent daily by farmer at the farm premises, (b) maximum temperature at 2 m, (c) relative humidity at 2 m
Vaccination against pneumonia – I & s-I – 2-year average	4	(a) Period spent daily by farmer at the farm premises, (c) relative humidity at 2 m
Vaccination against pneumonia – s-E & E – 10-year average	3	(a) Routine administration of antibiotics to newborns, (b) Period spent daily by farmer at the farm premises, (c) relative humidity at 2 m
Vaccination against pneumonia – s-E & E – 2-year average	3	(a) Routine administration of antibiotics to newborns, (b) Period spent daily by farmer at the farm premises, (c) relative humidity at 2 m
Vaccination against staphylococcal mastitis – sheep – 10-year average	3	(a) Collaboration with a veterinarian, (b) Use of laboratory diagnostic examinations in milk samples, (c) wind speed at 10 m
Vaccination against staphylococcal mastitis – sheep – 2-year average	3	(a) Collaboration with a veterinarian, (b) Use of laboratory diagnostic examinations in milk samples, (c) wind speed at 10 m
Vaccination against staphylococcal mastitis – goats – 10-year average	3	(a) Type of milking mode, (b) Daily number of milking sessions, (c) Daily period spent by farmer at the farm
Vaccination against staphylococcal mastitis – goats – 2-year average	4	(a) Type of milking mode, (b) Daily number of milking sessions, (c) Daily period spent by farmer at the farm
Vaccination against staphylococcal mastitis – I & s-I – 10-year average	5	(a) Type of milking mode, (b) Use of laboratory diagnostic examinations in milk samples, (c) Daily number of milking sessions
Vaccination against staphylococcal mastitis – I & s-I – 2-year average	5	(a) Type of milking mode, (b) Use of laboratory diagnostic examinations in milk samples, (c) Daily number of milking sessions
Vaccination against staphylococcal mastitis – s-E & E – 10-year average	7	(a) Type of milking mode, (b) Collaboration with a veterinarian, (c) Use of laboratory diagnostic examinations in milk samples, (d) Daily number of milking sessions

Vaccination against staphylococcal mastitis – s-E & E – 2-year average	9	(a) Type of milking mode, (b) Collaboration with a veterinarian, (c) temperature of Earth skin, (d) temperature at 2 m, (e) precipitation, (f) wind speed at 10 m
Total number of optional vaccines administered – sheep – 10-year average	10	(a) Management system applied in farms, (b) Collaboration with a veterinarian, (c) Use of laboratory diagnostic examinations in milk samples, (d) Age of newborns when taken away from dam, (e) Daily number of milking sessions, (f) Routine administration of antibiotics to newborns, (g) Age of farmers,
Total number of optional vaccines administered – sheep – 2-year average	11	(a) Management system applied in farms, (b) Collaboration with a veterinarian, (c) Use of laboratory diagnostic examinations in milk samples, (d) Age of newborns when taken away from dam, (e) Daily number of milking sessions, (f) Routine administration of antibiotics to newborns, (g) Age of farmers, (h) temperature of Earth skin, (i) maximum temperature at 2 m, (j) minimum temperature at 2 m
Total number of optional vaccines administered – goats – 10-year average	2	(a) Period spent daily by farmer at the farm premises, (b) maximum temperature at 2 m
Total number of optional vaccines administered – goats – 2-year average	1	(a) Period spent daily by farmer at the farm premises
Total number of optional vaccines administered – I & s-I – 10-year average	7	(a) Use of laboratory diagnostic examinations in milk samples, (b) Age of newborns when taken away from dam, (c) Daily number of milking sessions
Total number of optional vaccines administered – I & s-I – 2-year average	7	(a) Use of laboratory diagnostic examinations in milk samples, (b) Age of newborns when taken away from dam, (c) Daily number of milking sessions
Total number of optional vaccines administered – s-E & E – 10-year average	11	(a) Collaboration with a veterinarian, (b) Use of laboratory diagnostic examinations in milk samples, (c) Age of newborns when taken away from dam, (d) Routine administration of antibiotics to newborns, (e) Age of farmers, (f) Period spent daily by farmer at the farm premises, (g) temperature at 2 m, (h) maximum temperature at 2 m, (i) temperature range at 2 m, (j) precipitation
Total number of optional vaccines administered – s-E & E – 2-year average	11	(a) Collaboration with a veterinarian, (b) Use of laboratory diagnostic examinations in milk samples, (c) Age of newborns when taken away from dam, (d) Daily number of milking sessions, (e) Routine administration of antibiotics to newborns, (e) Age of farmers, (f) Period spent daily by farmer at the farm premises, (g) temperature at 2 m, (h) minimum temperature at 2 m, (i) wind speed at 10 m

1 I: intensive, E: extensive, s: semi.

Figure S1. Visual presentation of differences in the climatic variables assessed for associations with vaccination against infections in 444 small ruminant farms in Greece, with comparisons made between the 10-year (2010-2019) and the 2-year (2018-2019) datasets (average values for the entire time-period covered by each dataset): differences in shade between the two bars in the below histograms indicate significant differences between the respective values.

(a) Temperature of Earth skin (°C)

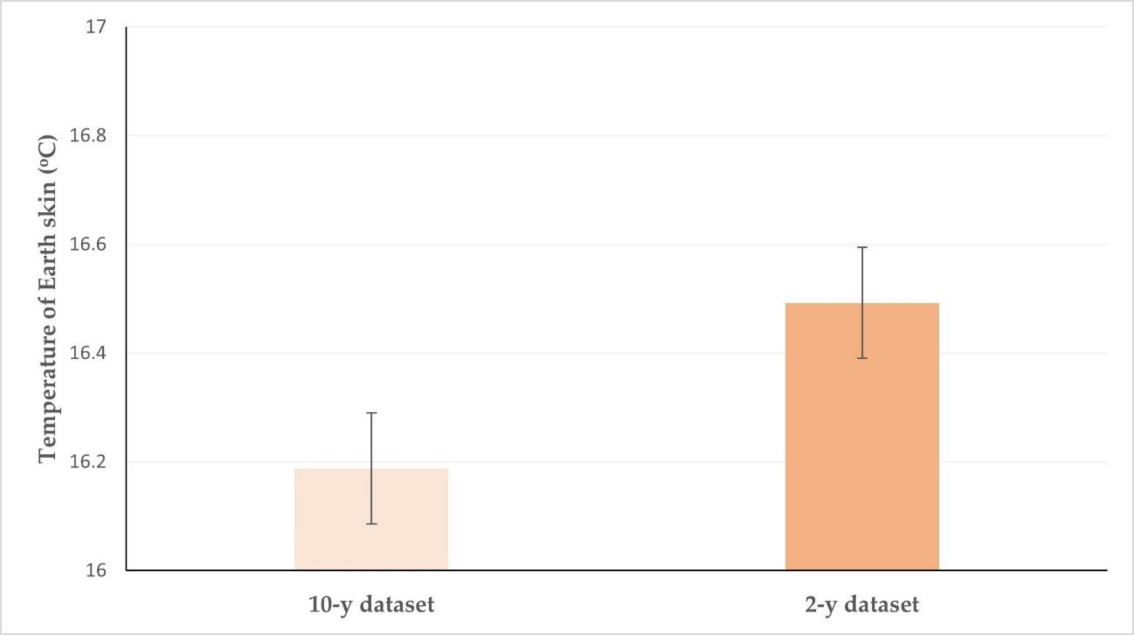

(b) Temperature at 2 m (°C)

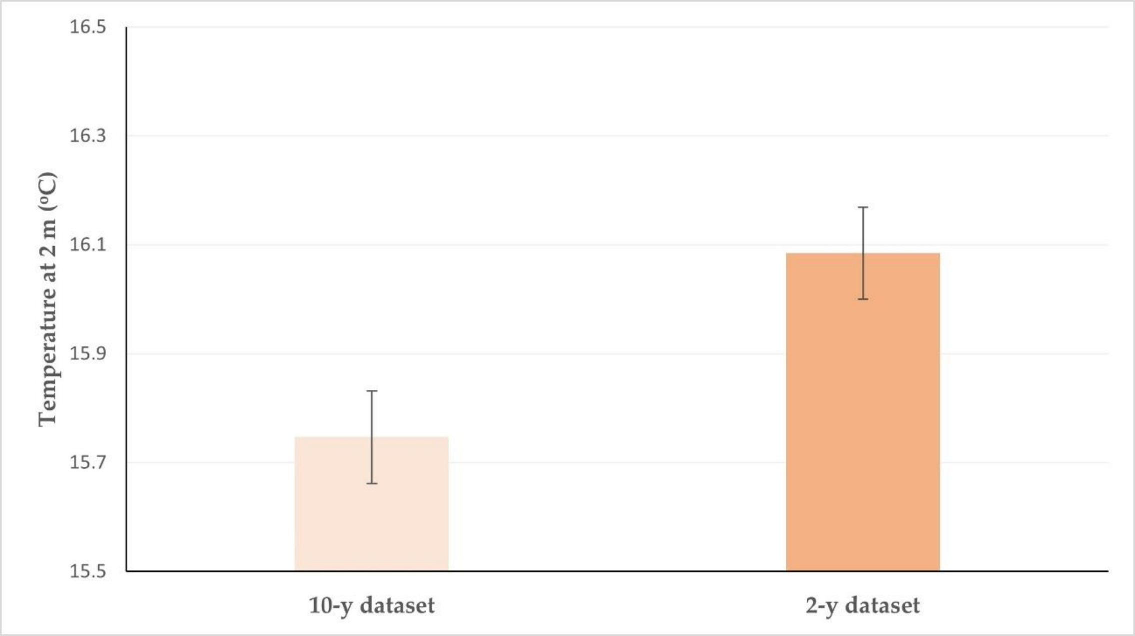

(c) Maximum temperature at 2 m (ºC)

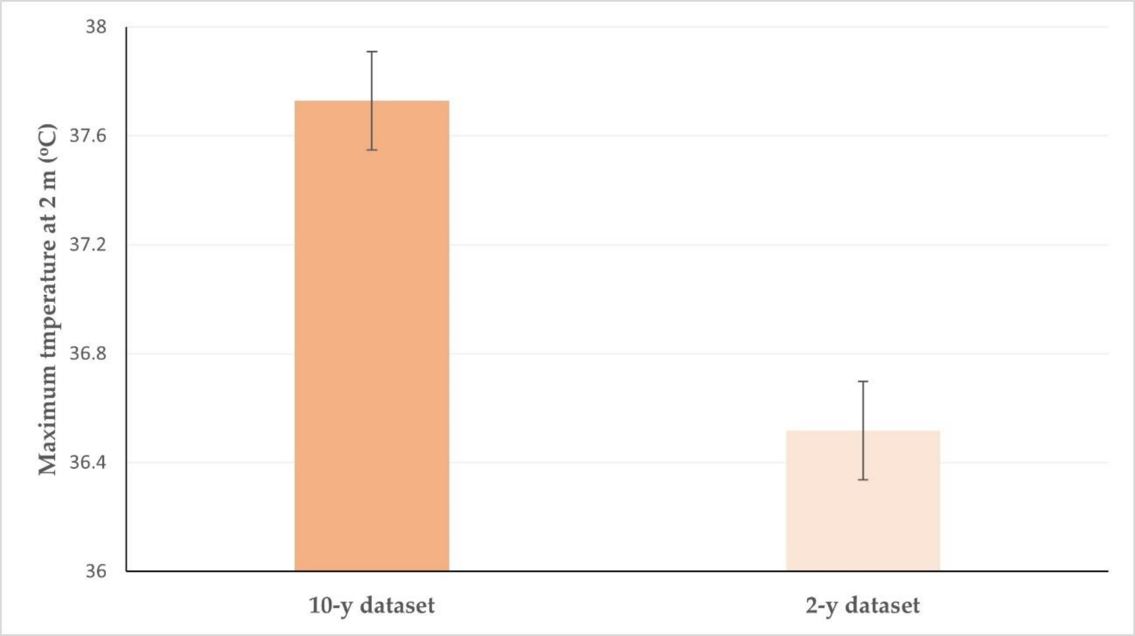

(d) Minimum temperature at 2 m (ºC)

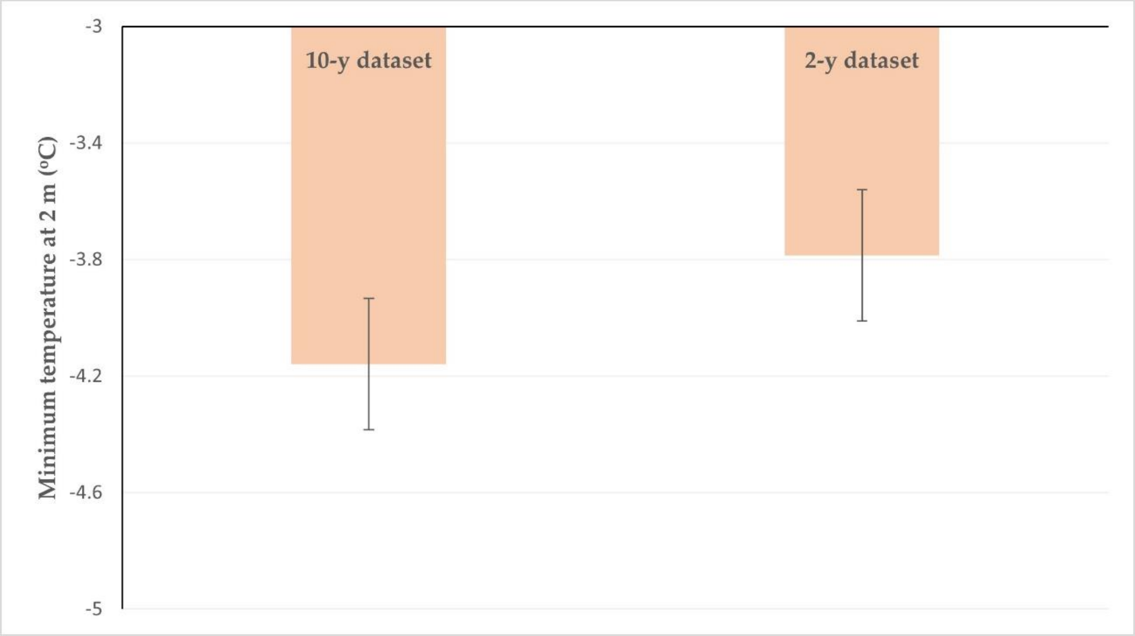

(e) Temperature range at 2 m (°C)

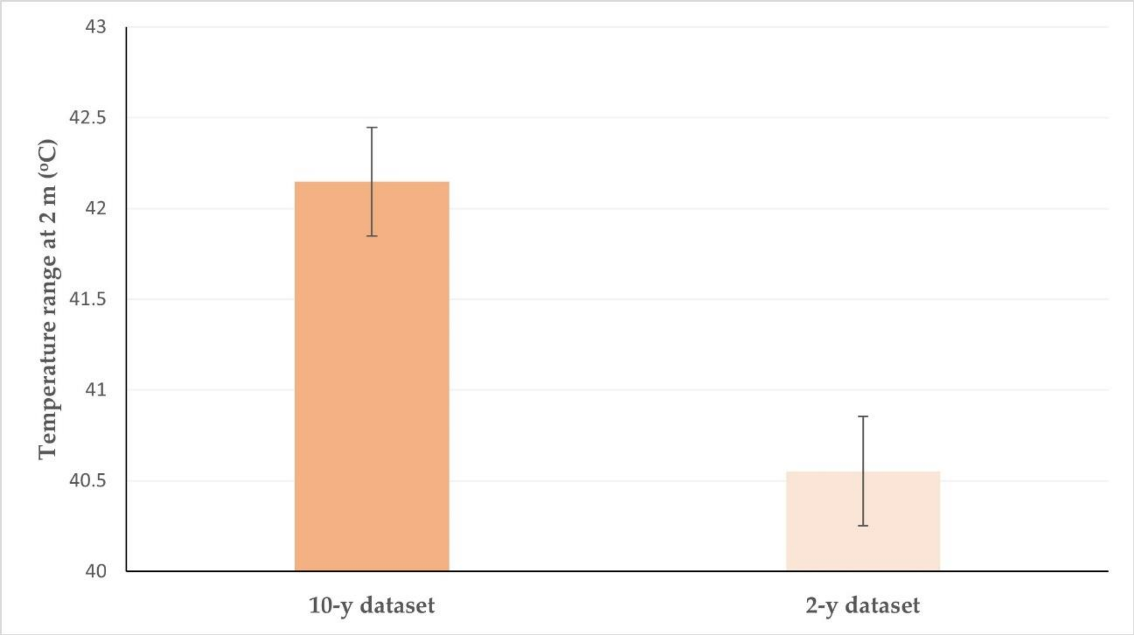

(f) Relative humidity at 2 m (%)

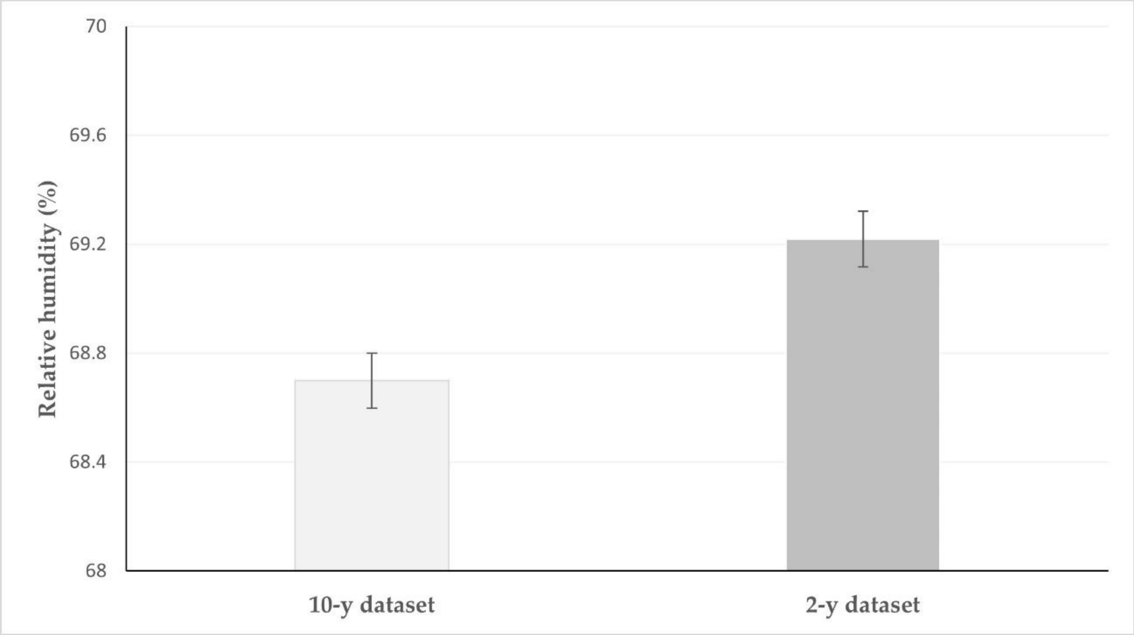

(g) Precipitation (mm)

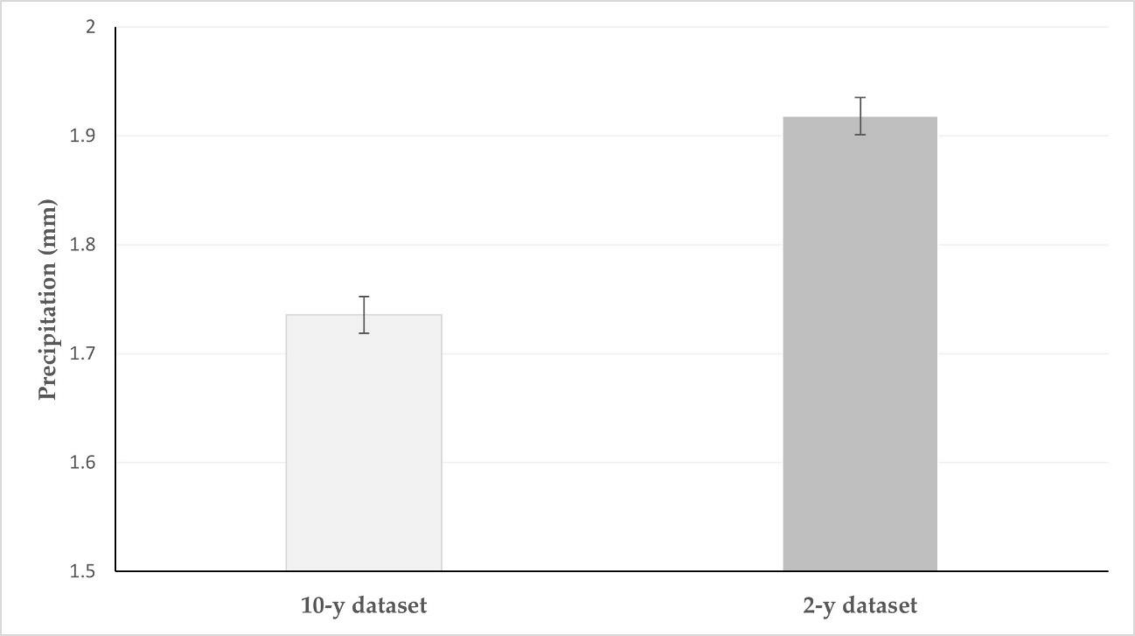

(h) Wind speed at 10 m (mm s^{-1})

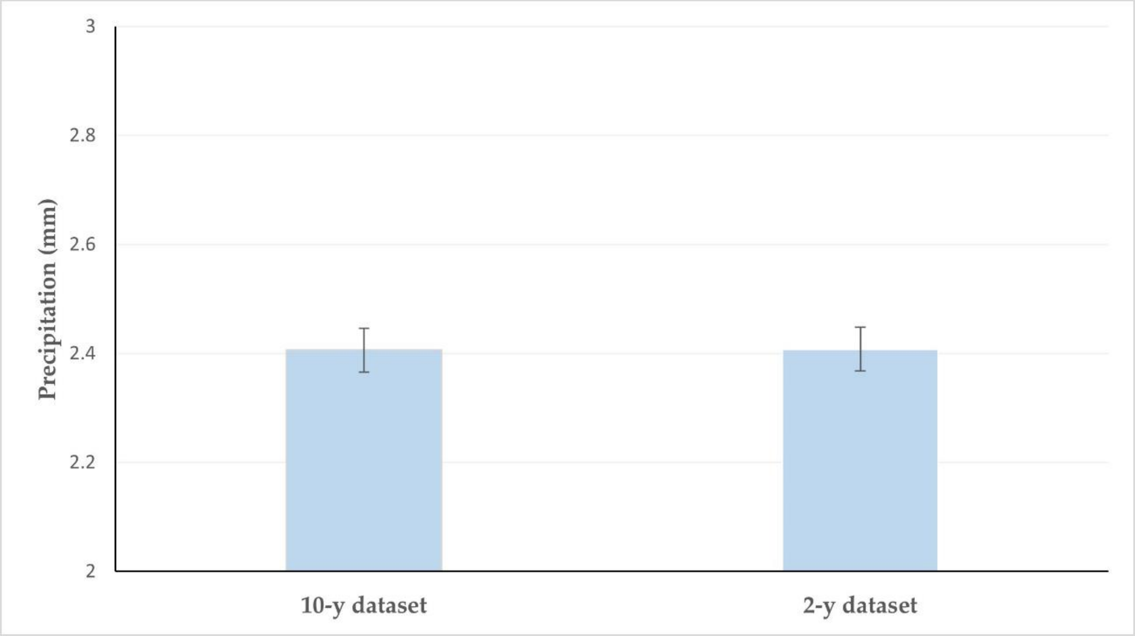

Table S3. Results of univariable analysis of climate conditions (mean ± standard error of the mean) for associations with optional vaccination against chlamydial abortion in 444 small ruminant farms in Greece.

Sheep flocks

**Vaccination performed**		**Vaccination not performed**		***p*** [1]
10-year average	2-year average	10-year average	2-year average	
		**Temperature of Earth skin (^{o}C)**		
15.8 ± 0.2	16.2 ± 0.2	16.5 ± 0.2	16.8 ± 0.2	0.007 / 0.007
		**Temperature at 2 m (^{o}C)**		
15.5 ± 0.1	15.8 ± 0.1	16.0 ± 0.1	16.3 ± 0.2	0.019 / 0.017
		**Maximum temperature at 2 m (^{o}C)**		
37.9 ± 0.5	39.6 ± 0.5	37.7 ± 0.2	36.5 ± 0.2	0.72 / 0.81
		**Minimum temperature at 2 m (^{o}C)**		
–4.7 ± 0.4	–0.5 ± 0.4	–3.6 ± 0.3	–0.2 ± 0.4	0.024 / 0.015
		**Temperature range at 2 m (^{o}C)**		
43.5 ± 0.2	41.9 ± 0.4	41.3 ± 0.5	39.7 ± 0.5	0.0005 / 0.0006
		**Relative humidity at 2 m (%)**		
68.4 ± 0.2	68.9 ± 0.2	68.8 ± 0.2	69.3 ± 0.2	0.10 / 0.08
		**Precipitation (mm)**		
1.8 ± 0.0	2.0 ± 0.0	1.7 ± 0.0	1.9 ± 0.0	0.08 / 0.13
		**Wind speed at 10 m (m s^{-1})**		
2.2± 0.1	2.2 ± 0.1	2.5 ± 0.1	2.5 ± 0.1	<0.0001 / < 0.0001

Goat herds

**Vaccination performed**		**Vaccination not performed**		***p***
10-year average	2-year average	10-year average	2-year average	
		**Temperature of Earth skin (^{o}C)**		
15.6 ± 0.3	15.9 ± 0.3	16.3 ± 0.3	16.7 ± 0.3	0.06 / 0.06
		**Temperature at 2 m (^{o}C)**		
15.3 ± 0.3	15.6 ± 0.3	15.9 ± 0.2	16.2 ± 0.2	0.10 / 0.09
		**Maximum temperature at 2 m (^{o}C)**		
38.3 ± 0.3	36.9 ± 0.3	37.4 ± 0.3	36.3 ± 0.3	0.045 / 0.09

		**Minimum temperature at 2 m (°C)**		
–0.4 ± 0.7	–0.2 ± 0.7	–0.8 ± 0.6	–0.4 ± 0.6	0.08 / 0.08
		**Temperature range at 2 m (°C)**		
43.7 ± 0.9	42.1 ± 0.9	41.2 ± 0.8	39.7 ± 0.8	0.035 / 0.043
		**Relative humidity at 2 m (%)**		
68.9 ± 0.3	69.3 ± 0.3	68.9 ± 0.2	69.4 ± 0.2	0.85 / 0.86
		**Precipitation (mm)**		
1.8 ± 0.1	2.0 ± 0.1	1.7 ± 0.0	1.9 ± 0.0	0.019 / 0.09
		**Wind speed at 10 m (m s^{-1})**		
2.2± 0.1	2.2 ± 0.1	2.6 ± 0.1	2.6 ± 0.1	0.011 / 0.010

Farms with intensive or semi-intensive management

**Vaccination performed**		**Vaccination not performed**		***p***
10-year average	2-year average	10-year average	2-year average	
		**Temperature of Earth skin (°C)**		
15.5 ± 0.2	16.0 ± 0.2	15.7 ± 0.2	15.8 ± 0.2	0.38 / 0.42
		**Temperature at 2 m (°C)**		
15.2 ± 0.1	15.8 ± 0.1	15.4 ± 0.1	15.6 ± 0.1	0.34 / 0.34
		**Maximum temperature at 2 m (°C)**		
37.9 ± 0.6	37.2 ± 0.2	38.5 ± 0.3	36.7 ± 0.6	0.34 / 0.40
		**Minimum temperature at 2 m (°C)**		
–5.5 ± 0.4	–5.3 ± 0.4	–5.4 ± 0.8	–5.3 ± 0.4	0.84 / 0.99
		**Temperature range at 2 m (°C)**		
44.6 ± 0.4	42.5 ± 0.5	44.0 ± 0.3	43.0 ± 0.4	0.31 / 0.41
		**Relative humidity at 2 m (%)**		
68.2 ± 0.2	68.5 ± 0.2	68.1 ± 0.6	68.6 ± 0.2	0.55 / 0.82
		**Precipitation (mm)**		
1.7 ± 0.0	1.9 ± 0.0	1.7 ± 0.8	1.9 ± 0.0	0.77 / 0.60
		**Wind speed at 10 m (m s^{-1})**		
2.1± 0.1	2.1 ± 0.1	2.1 ± 0.4	2.0 ± 0.1	0.37 / 0.37

Farms with semi-extensive or extensive management

Vaccination performed		Vaccination not performed		p
10-year average	2-year average	10-year average	2-year average	
		**Temperature of Earth skin (°C)**		
16.3 ± 0.3	16.5 ± 0.2	17.0 ± 0.2	17.3 ± 0.2	0.023 / 0.018
		**Temperature at 2 m (°C)**		
15.8 ± 0.2	16.1 ± 0.2	16.3 ± 0.2	16.6 ± 0.2	0.07 / 0.06
		**Maximum temperature at 2 m (°C)**		
38.0 ± 0.3	36.7 ± 0.3	36.9 ± 0.2	35.8 ± 0.2	0.001 / 0.010
		**Minimum temperature at 2 m (°C)**		
–3.9 ± 0.5	–3.6 ± 0.6	–2.4 ± 0.4	–1.7 ± 0.4	0.025 / 0.011
		**Temperature range at 2 m (°C)**		
41.9 ± 0.7	40.2 ± 0.7	39.2 ± 0.6	37.5 ± 0.6	0.004 / 0.004
		**Relative humidity at 2 m (%)**		
68.9 ± 0.2	69.6 ± 0.2	69.4 ± 0.2	70.0 ± 0.2	0.19 / 0.24
		**Precipitation (mm)**		
1.9 ± 0.0	2.1 ± 0.1	1.7 ± 0.0	1.9 ± 0.0	0.0001 / < 0.0001
		**Wind speed at 10 m (m s^{-1})**		
2.4± 0.1	2.4 ± 0.1	2.9 ± 0.1	2.9 ± 0.1	0.0002 / 0.0001

1. xxx / zzz: *p*-value for comparison between farms in which vaccination was or was not performed for 10-year average / 2-year average

Table S4. Results of univariable analysis of climate conditions (mean ± standard error of the mean) for associations with optional vaccination against clostridial infections in 444 small ruminant farms in Greece.

Sheep flocks

**Vaccination performed**		**Vaccination not performed**		***p*** [1]
10-year average	2-year average	10-year average	2-year average	
		**Temperature of Earth skin (^{o}C)**		
16.2 ± 0.1	16.2 ± 0.1	18.2 ± 0.9	18.2 ± 0.9	0.05 / 0.05
		**Temperature at 2 m (^{o}C)**		
15.7 ± 0.1	15.7 ± 0.1	16.9 ± 0.6	17.0 ± 0.1	0.07 / 0.07
		**Maximum temperature at 2 m (^{o}C)**		
37.8 ± 0.2	37.7 ± 0.2	36.2 ± 0.9	36.5 ± 0.9	0.14 / 0.14
		**Minimum temperature at 2 m (^{o}C)**		
–0.2 ± 0.3	–0.2 ± 0.3	–0.1 ± 1.8	–0.1 ± 1.8	0.048 / 0.049
		**Temperature range at 2 m (^{o}C)**		
42.4 ± 0.3	42.4 ± 0.3	36.2 ± 2.6	36.2 ± 2.6	0.049 / 0.049
		**Relative humidity at 2 m (%)**		
68.6 ± 0.1	68.6 ± 0.1	69.7 ± 0.7	69.7 ± 0.7	0.15 / 0.15
		**Precipitation (mm)**		
1.7 ± 0.0	1.7 ± 0.0	1.5 ± 0.1	1.5 ± 0.1	0.044 / 0.045
		**Wind speed at 10 m (m s^{-1})**		
2.4± 0.1	2.4 ± 0.1	3.3 ± 0.4	3.3 ± 0.4	0.044 / 0.045

Goat herds

**Vaccination performed**		**Vaccination not performed**		***p***
10-year average	2-year average	10-year average	2-year average	
		**Temperature of Earth skin (^{o}C)**		
16.2 ± 0.1	16.4 ± 0.2	15.0	15.3	0.52 / 0.47
		**Temperature at 2 m (^{o}C)**		
15.7 ± 0.1	16.0 ± 0.2	14.5	14.7	0.41 / 0.35
		**Maximum temperature at 2 m (^{o}C)**		
37.8 ± 0.2	36.5 ± 0.2	37.5	35.7	0.94 / 0.64
		**Minimum temperature at 2 m (^{o}C)**		
–0.2 ± 0.3	–0.0 ± 0.5	–0.6	–0.9	0.94/ 0.97

**Temperature range at 2 m (°C)**				
42.4 ± 0.3	40.5 ± 0.6	42.2	39.6	0.97 / 0.85
**Relative humidity at 2 m (%)**				
68.6 ± 0.1	69.4 ± 0.2	71.4	72.3	0.049 / 0.035
**Precipitation (mm)**				
1.7 ± 0.0	1.9 ± 0.0	2.0	2.3	0.21 / 0.15
**Wind speed at 10 m (m s⁻¹)**				
2.4± 0.1	2.4 ± 0.1	2.3	2.4	0.87 / 0.09

Farms with intensive or semi-intensive management

**Vaccination performed**		**Vaccination not performed**		***p***
10-year average	2-year average	10-year average	2-year average	
**Temperature of Earth skin (°C)**				
15.5 ± 0.2	15.9 ± 0.1	15.7 ± 0.2	15.7 ± 0.6	0.38 / 0.79
**Temperature at 2 m (°C)**				
15.2 ± 0.1	15.7 ± 0.1	15.4 ± 0.1	15.6 ± 0.4	0.34 / 0.86
**Maximum temperature at 2 m (°C)**				
37.9 ± 0.6	36.9 ± 0.3	38.5 ± 0.2	37.9 ± 0.9	0.34 / 0.48
**Minimum temperature at 2 m (°C)**				
–0.5 ± 0.4	–0.3 ± 0.3	–0.4 ± 0.3	–0.3 ± 2.0	0.84 / 0.99
**Temperature range at 2 m (°C)**				
44.6 ± 0.4	42.8 ± 0.3	44.0 ± 0.4	43.2 ± 0.9	0.31 / 0.0
**Relative humidity at 2 m (%)**				
68.2 ± 0.2	68.6 ± 0.1	68.1 ± 0.2	68.5 ± 0.5	0.55/ 0.90
**Precipitation (mm)**				
1.7 ± 0.0	1.9 ± 0.0	1.7 ± 0.1	2.0 ± 0.1	0.77 / 0.58
**Wind speed at 10 m (m s⁻¹)**				
2.1± 0.1	2.1 ± 0.1	2.1 ± 0.2	1.9 ± 0.2	0.37 / 0.59

Farms with semi-extensive or extensive management

Vaccination performed		Vaccination not performed		*p*
10-year average	2-year average	10-year average	2-year average	
**Temperature of Earth skin (°C)**				
16.3 ± 0.3	16.2 ± 0.1	17.0 ± 0.2	18.2 ± 0.9	0.05 / 0.05
**Temperature at 2 m (°C)**				
15.8 ± 0.2	15.7 ± 0.1	16.3 ± 0.2	17.0 ± 0.6	0.07 / 0.07
**Maximum temperature at 2 m (°C)**				
38.0 ± 0.3	37.8 ± 0.2	36.9 ± 0.2	36.2 ± 0.9	0.14 / 0.13
**Minimum temperature at 2 m (°C)**				
–0.9 ± 0.5	–0.2 ± 0.3	–0.4 ± 0.4	–0.1 ± 1.8	0.048 / 0.048
**Temperature range at 2 m (°C)**				
41.9 ± 0.7	42.4 ± 0.3	39.2 ± 0.6	36.2 ± 2.6	0.049 / 0.049
**Relative humidity at 2 m (%)**				
68.9 ± 0.2	68.6 ± 0.1	69.4 ± 0.2	70.0 ± 0.7	0.15 / 0.15
**Precipitation (mm)**				
1.9 ± 0.1	1.7 ± 0.0	1.7 ± 0.0	1.5 ± 0.1	0.045 / 0.045
**Wind speed at 10 m (m s^{-1})**				
2.4± 0.1	2.4 ± 0.1	2.9 ± 0.1	3.2 ± 0.4	0.045 / 0.044

1. xxx / zzz: p-value for comparison between farms in which vaccination was or was not performed for 10-year average / 2-year average

Table S5. Results of univariable analysis of climate conditions (mean ± standard error of the mean) for associations with optional vaccination against contagious agalactia in 444 small ruminant farms in Greece.

Sheep flocks

Vaccination performed		Vaccination not performed		p [1]
10-year average	2-year average	10-year average	2-year average	
**Temperature of Earth skin (°C)**				
15.7 ± 0.1	16.0 ± 0.1	17.0 ± 0.2	17.3 ± 0.2	< 0.0001 / < 0.0001
**Temperature at 2 m (°C)**				
15.3 ± 0.1	15.7 ± 0.1	16.4 ± 0.2	16.7 ± 0.2	< 0.0001 / < 0.0001
**Maximum temperature at 2 m (°C)**				
38.3 ± 0.3	37.0 ± 0.3	37.1 ± 0.4	36.0 ± 0.4	0.013 / 0.036
**Minimum temperature at 2 m (°C)**				
–0.2 ± 0.3	–0.9 ± 0.3	–0.5 ± 0.4	–0.1 ± 0.5	< 0.0001 / 0.049
**Temperature range at 2 m (°C)**				
43.8 ± 0.3	42.2 ± 0.3	40.0 ± 0.6	38.4 ± 0.6	< 0.0001 / < 0.0001
**Relative humidity at 2 m (%)**				
68.4 ± 0.0	68.9 ± 0.2	68.9 ± 0.2	69.4 ± 0.2	0.035 / < 0.0001
**Precipitation (mm)**				
1.8 ± 0.0	1.9 ± 0.0	1.7 ± 0.0	1.9 ± 0.1	0.15 / 0.06
**Wind speed at 10 m (m s^{-1})**				
2.2± 0.1	2.2 ± 0.1	2.7 ± 0.1	2.7 ± 0.1	< 0.0001 / < 0.0001

Goat herds

Vaccination performed		Vaccination not performed		p
10-year average	2-year average	10-year average	2-year average	
**Temperature of Earth skin (°C)**				
15.6 ± 0.2	15.9 ± 0.2	16.7 ± 0.4	17.0 ± 0.4	0.016 / 0.013
**Temperature at 2 m (°C)**				
15.2 ± 0.2	15.6 ± 0.2	16.1 ± 0.3	16.5 ± 0.3	0.023 / 0.018
**Maximum temperature at 2 m (°C)**				
38.4 ± 0.2	37.1 ± 0.2	36.7 ± 0.4	35.7 ± 0.4	0.0006 / 0.0007
**Minimum temperature at 2 m (°C)**				
–0.5 ± 0.5	–0.3 ± 0.5	–0.9 ± 0.8	–0.4 ± 0.8	0.007/ 0.005

**Temperature range at 2 m (°C)**				
43.9 ± 0.6	42.4 ± 0.5	39.7 ± 1.1	38.1 ± 1.1	0.0009 / 0.0007
**Relative humidity at 2 m (%)**				
68.5 ± 0.2	68.9 ± 0.2	69.4 ± 0.2	69.9 ± 0.2	0.008 / 0.006
**Precipitation (mm)**				
1.7 ± 0.0	1.9 ± 0.0	1.7 ± 0.1	1.9 ± 0.1	0.85 / 0.87
**Wind speed at 10 m (m s⁻¹)**				
2.2± 0.1	2.2 ± 0.1	2.7 ± 0.1	2.8 ± 0.1	0.001 / 0.001

Farms with intensive or semi-intensive management

**Vaccination performed**		**Vaccination not performed**		***p***
10-year average	2-year average	10-year average	2-year average	
**Temperature of Earth skin (°C)**				
15.4 ± 0.2	15.7 ± 0.1	16.0 ± 0.3	16.3 ± 0.3	0.027 / 0.026
**Temperature at 2 m (°C)**				
15.2 ± 0.1	15.5 ± 0.1	15.6 ± 0.2	16.0 ± 0.2	0.05 / 0.05
**Maximum temperature at 2 m (°C)**				
38.6 ± 0.3	37.3 ± 0.3	37.6 ± 0.7	36.3 ± 0.6	0.21 / 0.20
**Minimum temperature at 2 m (°C)**				
–0.0 ± 0.3	–0.9 ± 0.3	–0.5 ± 0.6	–0.1 ± 0.6	0.014 / 0.011
**Temperature range at 2 m (°C)**				
44.9 ± 0.3	43.5 ± 0.3	42.9 ± 0.6	41.2 ± 0.7	0.004 / 0.004
**Relative humidity at 2 m (%)**				
68.0 ± 0.1	68.4 ± 0.2	68.4 ± 0.3	69.0 ± 0.3	0.15 / 0.09
**Precipitation (mm)**				
1.7 ± 0.0	1.9 ± 0.0	1.7 ± 0.0	1.9 ± 0.1	0.55 / 0.80
**Wind speed at 10 m (m s⁻¹)**				
2.0± 0.0	2.0 ± 0.1	2.3 ± 0.1	2.3 ± 0.1	0.001 / 0.0009

Farms with semi-extensive or extensive management

Vaccination performed		Vaccination not performed		*p*
10-year average	2-year average	10-year average	2-year average	
**Temperature of Earth skin (°C)**				
16.0 ± 0.2	16.3 ± 0.2	17.4 ± 0.2	17.7 ± 0.2	< 0.0001 / < 0.0001
**Temperature at 2 m (°C)**				
15.6 ± 0.2	15.9 ± 0.2	16.7 ± 0.2	17.0 ± 0.2	< 0.0001 / < 0.0001
**Maximum temperature at 2 m (°C)**				
37.9 ± 0.2	36.6 ± 0.2	36.6 ± 0.3	35.6 ± 0.3	0.0001 / 0.002
**Minimum temperature at 2 m (°C)**				
–0.3 ± 0.4	–0.8 ± 0.4	–1.6 ± 0.5	–1.0 ± 0.5	< 0.0001 / < 0.0001
**Temperature range at 2 m (°C)**				
42.2 ± 0.5	40.4 ± 0.4	38.2 ± 0.7	36.6 ± 0.7	< 0.0001 / < 0.0001
**Relative humidity at 2 m (%)**				
69.1 ± 0.2	69.8 ± 0.2	69.4 ± 0.2	70.0 ± 0.2	0.18 / 0.48
**Precipitation (mm)**				
1.8 ± 0.0	2.0 ± 0.0	1.7 ± 0.0	1.9 ± 0.1	0.18 / 0.06
**Wind speed at 10 m (m s^{-1})**				
2.4± 0.1	2.4 ± 0.1	3.0 ± 0.1	3.0 ± 0.1	< 0.0001 / < 0.0001

1. xxx / zzz: p-value for comparison between farms in which vaccination was or was not performed for 10-year average / 2-year average

Table S6. Results of univariable analysis of climate conditions (mean ± standard error of the mean) for associations with optional vaccination against contagious ecthyma in 444 small ruminant farms in Greece.

Sheep flocks

Vaccination performed		Vaccination not performed		*p* [1]
10-year average	2-year average	10-year average	2-year average	
**Temperature of Earth skin (°C)**				
12.9 ± 1.6	13.5 ± 1.5	16.2 ± 0.2	16.5 ± 0.1	0.17 / 0.18
**Temperature at 2 m (°C)**				
12.8 ± 1.3	13.3 ± 1.4	15.8 ± 0.2	16.1 ± 0.1	0.16 / 0.18
**Maximum temperature at 2 m (°C)**				
35.9 ± 0.8	34.7 ± 0.9	37.8 ± 0.1	36.5 ± 0.2	0.14 / 0.17
**Minimum temperature at 2 m (°C)**				
–10.8 ± 2.5	–10.4 ± 3.1	–0.0 ± 0.1	–0.6 ± 0.3	0.11 / 0.16
**Temperature range at 2 m (°C)**				
46.7 ± 2.3	45.1 ± 2.6	42.2 ± 0.3	40.6 ± 0.3	0.18 / 0.23
**Relative humidity at 2 m (%)**				
69.6 ± 0.2	69.3 ± 0.3	68.6 ± 0.1	69.2 ± 0.2	0.015 / 0.73
**Precipitation (mm)**				
1.6 ± 0.2	1.6 ± 0.1	1.7 ± 0.2	1.9 ± 0.0	0.22 / 0.13
**Wind speed at 10 m (m s^{-1})**				
2.0± 0.4	2.0 ± 0.4	2.4 ± 0.4	2.4 ± 0.1	0.38 / 0.44

Goat herds

Vaccination performed		Vaccination not performed		*p* [1]
10-year average	2-year average	10-year average	2-year average	
**Temperature of Earth skin (°C)**				
15.8	16.3	16.1 ± 0.2	16.4 ± 0.2	0.87 / 0.66
**Temperature at 2 m (°C)**				
15.4	15.9	15.7 ± 0.2	16.0 ± 0.2	0.83 / 0.60
**Maximum temperature at 2 m (°C)**				
36.1	35.1	37.7 ± 0.2	36.5 ± 0.2	0.39 / < 0.0001
**Minimum temperature at 2 m (°C)**				
–0.0	–0.8	–0.3 ± 0.5	–0.0± 0.5	0.64 / 0.13

		**Temperature range at 2 m (°C)**		
42.2	39.9	42.2 ± 0.6	40.5 ± 0.6	0.97 / 0.32
		**Relative humidity at 2 m (%)**		
69.4	69.7	68.9 ± 0.2	69.4 ± 0.2	0.72 / 0.13
		**Precipitation (mm)**		
1.7	1.8	1.7 ± 0.0	1.9 ± 0.0	0.83 / 0.001
		**Wind speed at 10 m (m s^{-1})**		
2.7	2.8	2.4 ± 0.1	2.4 ± 0.1	0.66 / < 0.001

Farms with intensive or semi-intensive management

**Vaccination performed**		**Vaccination not performed**		***p***
10-year average	2-year average	10-year average	2-year average	
		**Temperature of Earth skin (°C)**		
11.4 ± 1.0	12.0 ± 0.9	15.6 ± 0.1	15.9 ± 0.1	0.14 / 0.14
		**Temperature at 2 m (°C)**		
11.5 ± 0.8	12.0 ± 0.8	15.3 ± 0.1	15.7 ± 0.1	0.13 / 0.13
		**Maximum temperature at 2 m (°C)**		
35.8 ± 1.4	34.5 ± 1.5	38.3 ± 0.3	37.0 ± 0.3	0.32 / 0.34
		**Minimum temperature at 2 m (°C)**		
–13.2 ± 1.4	–13.3 ± 2.3	–0.4 ± 0.3	–0.2 ± 0.3	0.11 / 0.17
		**Temperature range at 2 m (°C)**		
48.9 ± 0.1	47.8 ± 0.7	44.2 ± 0.3	42.8 ± 0.3	< 0.0001 / < 0.0001
		**Relative humidity at 2 m (%)**		
69.8 ± 0.3	69.1 ± 0.3	68.1 ± 0.1	68.5 ± 0.1	0.044 / 0.33
		**Precipitation (mm)**		
1.6 ± 0.1	1.6 ± 0.1	1.7 ± 0.0	1.9 ± 0.0	0.41 / 0.24
		**Wind speed at 10 m (m s^{-1})**		
1.6± 0.1	1.6 ± 0.1	2.1 ± 0.1	2.1 ± 0.1	0.018 / 0.004

Farms with semi-extensive or extensive management

Vaccination performed		Vaccination not performed		*p*
10-year average	2-year average	10-year average	2-year average	
**Temperature of Earth skin (°C)**				
15.8	16.3	16.8 ± 0.2	17.1 ± 0.2	< 0.0001 / < 0.0001
**Temperature at 2 m (°C)**				
15.4	15.9	16.2 ± 0.1	16.5 ± 0.1	< 0.0001 / < 0.0001
**Maximum temperature at 2 m (°C)**				
36.1	35.1	37.2 ± 0.2	36.1 ± 0.2	< 0.0001 / < 0.0001
**Minimum temperature at 2 m (°C)**				
–0.0	–0.8	–0.8 ± 0.3	–0.2 ± 0.4	< 0.001 / < 0.0001
**Temperature range at 2 m (°C)**				
42.2	39.9	40.1 ± 0.5	38.3 ± 0.5	< 0.0001 / 0.001
**Relative humidity at 2 m (%)**				
69.4	69.7	69.3 ± 0.1	69.9 ± 0.1	0.50 / 0.14
**Precipitation (mm)**				
1.7	1.8	1.7 ± 0.0	2.0 ± 0.0	0.025 / < 0.0001
**Wind speed at 10 m (m s^{-1})**				
2.7	2.8	2.7 ± 0.1	2.7 ± 0.1	0.85 / 0.14

1. xxx / zzz: p-value for comparison between farms in which vaccination was or was not performed for 10-year average / 2-year average

Table S7. Results of univariable analysis of climate conditions (mean ± standard error of the mean) for associations with optional vaccination against foot-rot in 444 small ruminant farms in Greece.

Sheep flocks

Vaccination performed		Vaccination not performed		p [1]
10-year average	2-year average	10-year average	2-year average	
**Temperature of Earth skin (°C)**				
16.4 ± 0.6	16.7 ± 0.6	16.2 ± 0.7	16.5 ± 0.1	0.83 / < 0.0001
**Temperature at 2 m (°C)**				
15.9 ± 0.5	16.1 ± 0.5	15.8 ± 0.8	16.1 ± 0.1	0.98 / < 0.0001
**Maximum temperature at 2 m (°C)**				
38.0 ± 1.0	36.2 ± 1.1	37.7 ± 0.8	36.5 ± 0.2	0.80 / 0.036
**Minimum temperature at 2 m (°C)**				
–0.4 ± 1.1	–0.1 ± 1.4	–0.1 ± 0.6	–0.7 ± 0.3	0.67 / 0.049
**Temperature range at 2 m (°C)**				
41.4 ± 2.0	39.3 ± 2.4	42.2 ± 0.7	40.6 ± 0.3	0.63 / < 0.0001
**Relative humidity at 2 m (%)**				
69.3 ± 0.9	70.4 ± 0.9	68.6 ± 0.5	69.1 ± 0.1	0.25 / < 0.0001
**Precipitation (mm)**				
1.8 ± 0.1	2.1 ± 0.1	1.7 ± 0.5	1.9 ± 0.0	0.19 / 0.06
**Wind speed at 10 m (m s^{-1})**				
2.6± 0.4	2.6 ± 0.4	2.4 ± 0.6	2.4 ± 0.1	0.65 / < 0.0001

Goat herds

Farms with intensive or semi-intensive management

Vaccination performed		Vaccination not performed		p
10-year average	2-year average	10-year average	2-year average	
**Temperature of Earth skin (°C)**				
16.4 ± 0.7	16.7 ± 0.8	15.6 ± 0.1	15.9 ± 0.1	0.32 / 0.40
**Temperature at 2 m (°C)**				
15.9 ± 0.6	16.1 ± 0.7	15.6 ± 0.1	15.7 ± 0.1	0.45 / 0.56
**Maximum temperature at 2 m (°C)**				
37.8 ± 1.3	36.2 ± 1.4	38.3 ± 0.3	37.0 ± 0.3	0.75 / 0.59

**Minimum temperature at 2 m (°C)**				
–0.3 ± 1.4	–0.9 ± 1.8	–0.5 ± 0.3	–0.3 ± 0.3	0.21 / 0.27
**Temperature range at 2 m (°C)**				
41.1 ± 2.6	39.0 ± 3.1	44.3 ± 0.3	42.8 ± 0.3	0.30 / 0.31
**Relative humidity at 2 m (%)**				
69.6 ± 1.2	70.7 ± 1.1	68.1 ± 0.1	68.5 ± 0.1	0.28/ 0.15
**Precipitation (mm)**				
1.8 ± 0.1	2.1 ± 0.2	1.7 ± 0.0	1.9 ± 0.0	0.71 / 0.31
**Wind speed at 10 m (m s–1)**				
2.6± 0.5	2.6 ± 0.5	2.1 ± 0.1	2.1 ± 0.1	0.32 / 0.31

Farms with semi-extensive or extensive management

**Vaccination performed**		**Vaccination not performed**		***p***
10-year average	2-year average	10-year average	2-year average	
**Temperature of Earth skin (°C)**				
16.41	16.6	16.8 ± 0.2	17.1 ± 0.2	0.021 / 0.005
**Temperature at 2 m (°C)**				
15.93	16.1	16.2 ± 0.1	16.5 ± 0.1	0.05 / 0.004
**Maximum temperature at 2 m (°C)**				
38.63	36.6	37.2 ± 0.2	36.1 ± 0.2	< 0.0001 / < 0.0001
**Minimum temperature at 2 m (°C)**				
–0.9	–0.7	–0.8 ± 0.3	–0.3 ± 0.4	0.001 / < 0.0001
**Temperature range at 2 m (°C)**				
42.5	40.3	40.0 ± 0.5	38.3 ± 0.5	< 0.0001 / < 0.0001
**Relative humidity at 2 m (%)**				
68.4	69.3	69.3 ± 0.1	69.9 ± 0.1	< 0.0001 / < 0.0001
**Precipitation (mm)**				
1.9	2.3	1.7 ± 0.0	2.0 ± 0.0	< 0.0001 / 0.0001
**Wind speed at 10 m (m s–1)**				
2.3	2.3	2.7 ± 0.1	2.7 ± 0.1	< 0.0001 / < 0.0001

1. xxx / zzz: p-value for comparison between farms in which vaccination was or was not performed for 10-year average / 2-year average

Table S8. Results of univariable analysis of climate conditions (mean ± standard error of the mean) for associations with optional vaccination against paratuberculosis in 444 small ruminant farms in Greece.

Sheep flocks

Vaccination performed		Vaccination not performed		*p* [1]
10-year average	2-year average	10-year average	2-year average	
**Temperature of Earth skin (°C)**				
14.8 ± 0.7	14.8 ± 0.7	16.3 ± 0.1	16.3 ± 0.1	0.09 / 0.09
**Temperature at 2 m (°C)**				
14.5 ± 0.7	14.5 ± 0.7	15.8 ± 0.1	15.8 ± 0.1	0.08 / 0.08
**Maximum temperature at 2 m (°C)**				
37.5 ± 0.6	37.5 ± 0.6	37.8 ± 0.7	37.8 ± 0.7	0.73 / 0.73
**Minimum temperature at 2 m (°C)**				
–0.6 ± 1.3	–0.5 ± 1.4	–0.0 ± 0.1	–0.0 ± 0.9	0.09 / 0.09
**Temperature range at 2 m (°C)**				
44.1 ± 1.0	44.1 ± 1.0	42.1 ± 0.1	42.1 ± 0.3	0.09 / 0.09
**Relative humidity at 2 m (%)**				
69.5 ± 0.8	69.5 ± 0.8	68.6 ± 0.3	68.6 ± 0.1	0.28 / 0.28
**Precipitation (mm)**				
1.8 ± 0.1	1.8 ± 0.1	1.7 ± 0.8	1.7 ± 0.0	0.83 / 0.83
**Wind speed at 10 m (m s^{-1})**				
2.3± 0.2	2.3 ± 0.2	2.4 ± 0.4	2.4 ± 0.4	0.43 / 0.43

Goat herds

Vaccination performed		Vaccination not performed		*p*
10-year average	2-year average	10-year average	2-year average	
**Temperature of Earth skin (°C)**				
15.9 ± 0.4	15.9 ± 0.4	16.2 ± 0.3	16.2 ± 0.3	0.64 / 0.64
**Temperature at 2 m (°C)**				
15.5 ± 0.4	15.5 ± 0.4	15.7 ± 0.2	15.7 ± 0.2	0.57 / 0.57
**Maximum temperature at 2 m (°C)**				
38.3 ± 0.4	38.3 ± 0.4	37.5 ± 0.3	37.5 ± 0.3	0.10 / 0.10
**Minimum temperature at 2 m (°C)**				
–0.8 ± 0.9	–0.8 ± 0.9	–0.2 ± 0.6	–0.1 ± 0.6	0.52/ 0.52

**Temperature range at 2 m (°C)**				
43.1 ± 1.0	43.1 ± 1.0	41.6 ± 0.8	41.6 ± 0.8	0.26 / 0.26
**Relative humidity at 2 m (%)**				
68.6 ± 0.3	68.6 ± 0.3	69.0 ± 0.2	69.0 ± 0.2	0.30 / 0.30
**Precipitation (mm)**				
1.7 ± 0.1	2.3 ± 0.1	1.7 ± 0.0	1.7 ± 0.0	0.79 / 0.79
**Wind speed at 10 m (m s⁻¹)**				
2.3± 0.1	2.2 ± 0.1	2.5 ± 0.1	2.5 ± 0.1	0.42 / 0.42

Farms with intensive or semi-intensive management

**Vaccination performed**		**Vaccination not performed**		***p***
10-year average	2-year average	10-year average	2-year average	
**Temperature of Earth skin (°C)**				
14.1 ± 0.6	15.7 ± 0.1	14.1 ± 0.6	15.7 ± 0.1	0.013 / 0.013
**Temperature at 2 m (°C)**				
14.0 ± 0.5	15.5 ± 0.1	14.0 ± 0.5	15.5 ± 0.1	0.013 / 0.013
**Maximum temperature at 2 m (°C)**				
37.9 ± 0.6	37.3 ± 0.3	37.9 ± 0.6	37.3 ± 0.3	0.56 / 0.56
**Minimum temperature at 2 m (°C)**				
–0.9 ± 0.9	–0.9 ± 0.3	–0.9 ± 0.9	–0.9 ± 0.3	0.001 / 0.001
**Temperature range at 2 m (°C)**				
46.7 ± 0.7	43.5 ± 0.3	46.7 ± 0.7	43.5 ± 0.3	0.002 / 0.002
**Relative humidity at 2 m (%)**				
68.7 ± 0.4	68.4 ± 0.2	68.7 ± 0.4	68.4 ± 0.2	0.19 / 0.19
**Precipitation (mm)**				
1.7 ± 0.1	1.9 ± 0.0	1.7 ± 0.1	1.9 ± 0.0	0.30 / 0.30
**Wind speed at 10 m (m s⁻¹)**				
2.0± 0.1	2.0 ± 0.1	2.0± 0.1	2.0 ± 0.1	0.37 / 0.37

Farms with semi-extensive or extensive management

Vaccination performed		Vaccination not performed		*p*
10-year average	2-year average	10-year average	2-year average	
**Temperature of Earth skin (°C)**				
16.6 ± 0.4	16.3 ± 0.2	16.6 ± 0.4	16.8 ± 0.2	0.68 / 0.68
**Temperature at 2 m (°C)**				
16.0 ± 0.3	15.9 ± 0.2	16.0 ± 0.3	16.2 ± 0.1	0.61 / 0.61
**Maximum temperature at 2 m (°C)**				
38.2 ± 0.4	36.6 ± 0.2	38.2 ± 0.4	37.1 ± 0.2	0.010 / 0.010
**Minimum temperature at 2 m (°C)**				
–0.1 ± 0.8	–0.8 ± 0.4	–0.1 ± 0.8	–0.8 ± 0.4	0.73 / 0.73
**Temperature range at 2 m (°C)**				
41.3 ± 1.0	40.4 ± 0.4	41.3 ± 1.0	39.9 ± 0.5	0.22 / 0.22
**Relative humidity at 2 m (%)**				
68.9 ± 0.5	69.8 ± 0.2	68.9 ± 0.5	69.3 ± 0.1	0.39 / 0.39
**Precipitation (mm)**				
1.8 ± 0.1	2.0 ± 0.0	1.8 ± 0.1	1.7 ± 0.0	0.52/ 0.52
**Wind speed at 10 m (m s^{-1})**				
2.5± 0.1	2.4 ± 0.1	2.5± 0.1	2.7 ± 0.2	0.18 / 0.18

1. xxx / zzz: p-value for comparison between farms in which vaccination was or was not performed for 10-year average / 2-year average

Table S9. Results of univariable analysis of climate conditions (mean ± standard error of the mean) for associations with optional vaccination against pneumonia in 444 small ruminant farms in Greece.

Sheep flocks

Vaccination performed		Vaccination not performed		*p* [1]
10-year average	2-year average	10-year average	2-year average	
**Temperature of Earth skin (°C)**				
16.3 ± 0.2	16.5 ± 0.2	16.2 ± 0.6	16.5 ± 0.9	0.64 / 0.82
**Temperature at 2 m (°C)**				
15.8 ± 0.1	16.1 ± 0.2	15.7 ± 0.7	16.1 ± 0.9	0.66 / 0.87
**Maximum temperature at 2 m (°C)**				
38.2 ± 0.2	36.9 ± 0.2	37.4 ± 0.6	36.2 ± 0.1	0.06 / 0.11
**Minimum temperature at 2 m (°C)**				
–0.9 ± 0.3	–0.5 ± 0.2	–0.2 ± 0.4	–0.9 ± 0.4	0.57 / 0.44
**Temperature range at 2 m (°C)**				
42.1 ± 0.5	40.4 ± 0.5	42.2 ± 0.4	40.7 ± 0.6	0.89 / 0.63
**Relative humidity at 2 m (%)**				
68.8 ± 0.2	69.5 ± 0.2	68.5 ± 0.5	68.9 ± 0.2	0.19 / 0.022
**Precipitation (mm)**				
1.7 ± 0.0	1.9 ± 0.0	1.7 ± 0.0	1.9 ± 0.0	0.91 / 0.25
**Wind speed at 10 m (m s^{-1})**				
2.4± 0.1	2.4 ± 0.1	2.4 ± 0.1	2.4 ± 0.1	0.75 / 0.75

Goat herds

Vaccination performed		Vaccination not performed		*p*
10-year average	2-year average	10-year average	2-year average	
**Temperature of Earth skin (°C)**				
16.5 ± 0.4	16.7 ± 0.4	15.9 ± 0.3	16.3 ± 0.2	0.27 / 0.34
**Temperature at 2 m (°C)**				
16.0 ± 0.4	16.2 ± 0.4	15.5 ± 0.2	15.9 ± 0.2	0.36 / 0.46
**Maximum temperature at 2 m (°C)**				
37.5 ± 0.5	36.4 ± 0.4	37.8 ± 0.3	36.5 ± 0.2	0.57 / 0.71
**Minimum temperature at 2 m (°C)**				
–0.2 ± 0.9	–0.7 ± 1.0	–0.9 ± 0.3	–0.6 ± 0.6	0.12/ 0.08

**Temperature range at 2 m (°C)**				
40.7 ± 1.3	39.0 ± 1.3	42.7 ± 0.6	41.2 ± 0.7	0.18 / 0.14
**Relative humidity at 2 m (%)**				
69.5 ± 0.3	70.2 ± 0.3	68.6 ± 0.2	69.0 ± 0.2	0.013 / 0.003
**Precipitation (mm)**				
1.7 ± 0.0	1.9 ± 0.1	1.8 ± 0.0	1.9 ± 0.0	0.13 / 0.50
**Wind speed at 10 m (m s⁻¹)**				
2.7± 0.2	2.7 ± 0.2	2.3 ± 0.1	2.3 ± 0.1	0.07 / 0.08

Farms with intensive or semi-intensive management

**Vaccination performed**		**Vaccination not performed**		***p***
10-year average	2-year average	10-year average	2-year average	
**Temperature of Earth skin (°C)**				
16.0 ± 0.2	16.3 ± 0.2	15.3 ± 0.2	15.6 ± 0.2	0.0002 / 0.003
**Temperature at 2 m (°C)**				
15.7 ± 0.1	16.0 ± 0.1	15.1 ± 0.1	15.4 ± 0.1	0.004 / 0.006
**Maximum temperature at 2 m (°C)**				
38.8 ± 0.2	37.4 ± 0.2	37.9 ± 0.5	36.6 ± 0.5	0.11 / 0.15
**Minimum temperature at 2 m (°C)**				
–0.8 ± 0.3	–0.4 ± 0.4	–0.0 ± 0.4	–0.9 ± 0.4	0.020 / 0.009
**Temperature range at 2 m (°C)**				
43.6 ± 0.5	41.9 ± 0.4	44.8 ± 0.4	43.4 ± 0.4	0.05 / 0.023
**Relative humidity at 2 m (%)**				
68.3 ± 0.2	68.8 ± 0.2	68.0 ± 0.2	68.4 ± 0.2	0.36 / 0.11
**Precipitation (mm)**				
1.7 ± 0.0	1.9 ± 0.0	1.7 ± 0.0	1.9 ± 0.0	0.30 / 0.80
**Wind speed at 10 m (m s⁻¹)**				
2.2± 0.1	2.2 ± 0.1	2.0± 0.1	2.0 ± 0.1	0.029 / 0.027

Farms with semi-extensive or extensive management

Vaccination performed		Vaccination not performed		*p*
10-year average	2-year average	10-year average	2-year average	
**Temperature of Earth skin (°C)**				
16.6 ± 0.3	16.9 ± 0.3	16.9 ± 0.2	17.2 ± 0.2	0.49 / 0.32
**Temperature at 2 m (°C)**				
16.0 ± 0.2	16.3 ± 0.2	16.3 ± 0.2	16.6 ± 0.2	0.40 / 0.24
**Maximum temperature at 2 m (°C)**				
37.2 ± 0.3	36.1 ± 0.3	37.2 ± 0.2	36.0 ± 0.2	0.70 / 0.79
**Minimum temperature at 2 m (°C)**				
–0.6 ± 0.5	–0.1 ± 0.6	–0.9 ± 0.4	–0.4 ± 0.5	0.73 / 0.63
**Temperature range at 2 m (°C)**				
40.0 ± 0.8	38.2 ± 0.8	40.1 ± 0.6	38.4 ± 0.6	0.92 / 0.80
**Relative humidity at 2 m (%)**				
69.7 ± 0.2	70.5 ± 0.2	68.9 ± 0.2	69.5 ± 0.2	0.014 / 0.0005
**Precipitation (mm)**				
1.7 ± 0.0	2.0 ± 0.0	1.7 ± 0.0	1.9 ± 0.1	0.89/ 0.20
**Wind speed at 10 m (m s^{-1})**				
2.7± 0.1	2.8 ± 0.1	2.7± 0.1	2.7 ± 0.1	0.68 / 0.73

1. xxx / zzz: p-value for comparison between farms in which vaccination was or was not performed for 10-year average / 2-year average

Table S10. Results of univariable analysis of climate conditions (mean ± standard error of the mean) for associations with optional vaccination against staphylococcal mastitis in 444 small ruminant farms in Greece.

Sheep flocks

Vaccination performed		Vaccination not performed		*p* [1]
10-year average	2-year average	10-year average	2-year average	
		**Temperature of Earth skin (°C)**		
16.1 ± 0.2	16.4 ± 0.2	16.1 ± 0.2	16.5 ± 0.9	0.33 / 0.24
		**Temperature at 2 m (°C)**		
15.7 ± 0.1	16.0 ± 0.1	16.2 ± 0.1	16.1 ± 0.9	0.42 / 0.28
		**Maximum temperature at 2 m (°C)**		
38.2 ± 0.4	36.9 ± 0.4	36.3 ± 0.3	36.2 ± 0.1	0.10 / 0.19
		**Minimum temperature at 2 m (°C)**		
–0.3 ± 0.3	–0.0 ± 0.4	–0.5 ± 0.4	–0.9 ± 0.4	0.55 / 0.36
		**Temperature range at 2 m (°C)**		
43.0 ± 0.3	41.3 ± 0.5	40.1 ± 0.5	40.7 ± 0.6	0.049 / 0.06
		**Relative humidity at 2 m (%)**		
68.4 ± 0.2	69.0 ± 0.2	69.2 ± 0.2	68.9 ± 0.2	0.17 / 0.43
		**Precipitation (mm)**		
1.8 ± 0.0	2.0 ± 0.0	1.9 ± 0.0	1.9 ± 0.0	0.42 / 0.11
		**Wind speed at 10 m (m s^{-1})**		
2.2± 0.1	2.2 ± 0.1	2.5± 0.1	2.4 ± 0.1	0.008 / 0.007

Goat herds

Vaccination performed		Vaccination not performed		*p*
10-year average	2-year average	10-year average	2-year average	
		**Temperature of Earth skin (°C)**		
15.6 ± 0.4	15.9 ± 0.4	16.3 ± 0.3	16.6 ± 0.3	0.13 / 0.11
		**Temperature at 2 m (°C)**		
15.3 ± 0.3	15.6 ± 0.3	15.8 ± 0.2	16.2 ± 0.2	0.16 / 0.12
		**Maximum temperature at 2 m (°C)**		
38.1 ± 0.4	36.7 ± 0.4	37.5 ± 0.3	36.4 ± 0.3	0.23 / 0.60
		**Minimum temperature at 2 m (°C)**		
–0.2 ± 0.8	–0.0 ± 0.8	–0.0 ± 0.3	–0.6 ± 0.6	0.25/ 0.16

**Temperature range at 2 m (°C)**				
43.3 ± 1.1	41.7 ± 1.1	41.5 ± 0.6	40.0 ± 0.7	0.19 / 0.21
**Relative humidity at 2 m (%)**				
68.9 ± 0.3	69.5 ± 0.3	68.9 ± 0.2	69.3 ± 0.2	0.94 / 0.71
**Precipitation (mm)**				
1.8± 0.1	2.0 ± 0.1	1.7 ± 0.0	1.9 ± 0.1	0.08 / 0.07
**Wind speed at 10 m (m s⁻¹)**				
2.2± 0.1	2.2 ± 0.1	2.5 ± 0.1	2.5 ± 0.1	0.06 / 0.048

Farms with intensive or semi-intensive management

**Vaccination performed**		**Vaccination not performed**		***p***
10-year average	2-year average	10-year average	2-year average	
**Temperature of Earth skin (°C)**				
15.7 ± 0.1	16.0 ± 0.1	15.4 ± 0.2	15.8 ± 0.2	0.27 / 0.36
**Temperature at 2 m (°C)**				
15.4 ± 0.1	15.8 ± 0.1	15.2 ± 0.1	15.6 ± 0.2	0.24 / 0.34
**Maximum temperature at 2 m (°C)**				
38.5 ± 0.5	37.2 ± 0.5	38.01 ± 0.4	36.8 ± 0.4	0.43 / 0.56
**Minimum temperature at 2 m (°C)**				
–0.1 ± 0.3	–0.0 ± 0.3	–0.8 ± 0.4	–0.6 ± 0.4	0.18 / 0.24
**Temperature range at 2 m (°C)**				
44.2 ± 0.4	42.7 ± 0.4	44.3 ± 0.4	42.9 ± 0.5	0.89 / 0.76
**Relative humidity at 2 m (%)**				
68.1 ± 0.2	68.7 ± 0.2	68.2 ± 0.4	68.5 ± 0.2	0.78 / 0.56
**Precipitation (mm)**				
1.7 ± 0.0	2.0 ± 0.1	1.7 ± 0.0	1.8 ± 0.0	0.71 / 0.79
**Wind speed at 10 m (m s⁻¹)**				
2.1± 0.1	2.2 ± 0.1	2.1± 0.1	2.1 ± 0.1	0.78 / 0.027

Farms with semi-extensive or extensive management

Vaccination performed		Vaccination not performed		*p*
10-year average	2-year average	10-year average	2-year average	
**Temperature of Earth skin (°C)**				
16.4 ± 0.3	16.6 ± 0.3	17.2 ± 0.2	17.2 ± 0.2	0.16 / 0.11
**Temperature at 2 m (°C)**				
15.9 ± 0.3	16.1 ± 0.3	16.6 ± 0.1	16.6 ± 0.2	0.15 / 0.09
**Maximum temperature at 2 m (°C)**				
37.7 ± 0.3	36.3 ± 0.3	36.0 ± 0.2	36.0 ± 0.2	0.09 / 0.35
**Minimum temperature at 2 m (°C)**				
–0.3 ± 0.6	–0.0 ± 0.7	–0.0 ± 0.4	–0.4 ± 0.5	0.37 / 0.24
**Temperature range at 2 m (°C)**				
41.0 ± 0.9	39.2 ± 0.9	38.0 ± 0.6	38.4 ± 0.6	0.21 / 0.22
**Relative humidity at 2 m (%)**				
69.3 ± 0.3	69.9 ± 0.3	69.8 ± 0.2	69.5 ± 0.2	0.98 / 0.77
**Precipitation (mm)**				
1.8 ± 0.1	2.1 ± 0.1	1.9 ± 0.0	1.9 ± 0.1	0.06 / 0.012
**Wind speed at 10 m (m s^{-1})**				
2.5± 0.1	2.5 ± 0.1	2.8± 0.1	2.7 ± 0.1	0.11 / 0.026

1. xxx / zzz: p-value for comparison between farms in which vaccination was or was not performed for 10-year average / 2-year average

Table S11. Results of multivariable analysis of climate conditions for associations with optional vaccination against chlamydial abortion in 444 small ruminant farms in Greece.

Sheep flocks

10-year average		2-year average	
Variables	Regression coefficient (± standard error)	Variables	Regression coefficient (± standard error)
Wind speed at 10 m ($p = 0.0006$)	–0.262 ± 0.076	Wind speed at 10 m ($p = 0.0009$)	–0.261 ± 0.078

Goat herds

10-year average		2-year average	
Variables	Regression coefficient (± standard error)	Variables	Regression coefficient (± standard error)
Precipitation ($p = 0.013$)	0.328 ± 0.130	Wind speed at 10 m ($p = 0.018$)	–0.110 ± 0.046

Farms with intensive or semi-intensive management

Farms with semi-extensive or extensive management

10-year average		2-year average	
Variables	Regression coefficient (± standard error)	Variables	Regression coefficient (± standard error)
Wind speed at 10 m ($p < 0.0001$)	–0.468 ± 0.095	Precipitation ($p = 0.0009$)	0.251 ± 0.075
Temperature at 2 m ($p = 0.0001$)	0.145 ± 0.037	Wind speed at 10 m ($p = 0.047$)	–0.110 ± 0.046
Relative humidity at 2 m ($p = 0.0003$)	0.098 ± 0.027		

Table S12. Results of multivariable analysis of climate conditions for associations with optional vaccination against clostridial infections in 444 small ruminant farms in Greece.

Sheep flocks

10-year average		2-year average	
Variables	Regression coefficient (± standard error)	Variables	Regression coefficient (± standard error)
Temperature of Earth skin ($p < 0.0001$)	–0.306 ± 0.046	Temperature of Earth skin ($p < 0.0001$)	–0.248 ± 0.040
Temperature at 2 m ($p < 0.0001$)	0.347 ± 0.053	Temperature at 2 m ($p < 0.0001$)	0.278 ± 0.047
Relative humidity at 2 m ($p < 0.0001$)	0.027 ± 0.007	Relative humidity at 2 m ($p < 0.0001$)	0.018 ± 0.005
Precipitation ($p = 0.011$)	–0.087 ± 0.034		

Goat herds

Farms with intensive or semi-intensive management

Farms with semi-extensive or extensive management

10-year average	
Variables	Regression coefficient (± standard error)
Temperature at 2 m ($p < 0.0001$)	0.244 ± 0.052
Temperature of Earth skin ($p < 0.0001$)	–0.229 ± 0.050
Wind speed at 10 m ($p = 0.029$)	0.066 ± 0.030

Table S13. Results of multivariable analysis of climate conditions for associations with optional vaccination against contagious agalactia in 444 small ruminant farms in Greece.

Sheep flocks

10-year average		2-year average	
Variables	Regression coefficient (± standard error)	Variables	Regression coefficient (± standard error)
Wind speed at 10 m ($p < 0.0001$)	–0.240 ± 0.039	Wind speed at 10 m ($p < 0.0001$)	–0.257 ± 0.041
Relative humidity at 2 m ($p = 0.007$)	0.034 ± 0.015	Relative humidity at 2 m ($p = 0.007$)	0.041 ± 0.015

Goat herds

10-year average		2-year average	
Variables	Regression coefficient (± standard error)	Variables	Regression coefficient (± standard error)
Wind speed at 10 m ($p = 0.0002$)	–0.199 ± 0.052	Wind speed at 10 m ($p < 0.0001$)	–0.257 ± 0.016
		Relative humidity at 2 m ($p = 0.007$)	0.041 ± 0.015

Farms with intensive or semi-intensive management

10-year average		2-year average	
Variables	Regression coefficient (± standard error)	Variables	Regression coefficient (± standard error)
Wind speed at 10 m ($p < 0.0001$)	–0.203 ± 0.049	Wind speed at 10 m ($p = 0.009$)	–0.277 ± 0.105

Farms with semi-extensive or extensive management

10-year average		2-year average	
Variables	Regression coefficient (± standard error)	Variables	Regression coefficient (± standard error)
Temperature at 2 m ($p = 0.003$)	–0.179 ± 0.060	Temperature at 2 m ($p = 0.001$)	–0.170 ± 0.053

Maximum temperature at 2 m (p = 0.009)		Maximum temperature at 2 m (p = 0.024)	
	–0.179 ± 0.060		–0.076 ± 0.033
Temperature range at 2 m (p = 0.037)		Temperature range at 2 m (p = 0.042)	
	–0.054 ± 0.025		0.034 ± 0.017

Table S14. Results of multivariable analysis of climate conditions for associations with optional vaccination against contagious ecthyma in 444 small ruminant farms in Greece.

Sheep flocks

10-year average		2-year average	
Variables	Regression coefficient (± standard error)		
Minimum temperature at 2 m (p = 0.0005)	–0.012 ± 0.004	Minimum temperature at 2 m (p = 0.023)	–0.011 ± 0.005
Maximum temperature at 2 m (p = 0.014)	–0.004 ± 0.002	Maximum temperature at 2 m (p = 0.029)	–0.047 ± 0.002
Temperature range at 2 m (p = 0.018)	–0.007 ± 0.003	Temperature of Earth skin (p = 0.041)	0.045 ± 0.022

Goat herds

Farms with intensive or semi-intensive management

2-year average	
Variables	Regression coefficient (± standard error)
Temperature at 2 m (p = 0.032)	–0.081 ± 0.037

Farms with semi-extensive or extensive management

10-year average		2-year average	
Variables	Regression coefficient (± standard error)		
Maximum temperature at 2 m (p = 0.017)	–0.019 ± 0.008	Maximum temperature at 2 m (p = 0.038)	–4.234 ± 2.024
Temperature range at 2 m (p = 0.032)	0.013 ± 0.006	Minimum temperature at 2 m (p = 0.038)	4.222 ± 2.024
		Temperature range at 2 m (p = 0.038)	4.228 ± 2.024

Table S15. Results of multivariable analysis of climate conditions for associations with optional vaccination against foot-rot in 444 small ruminant farms in Greece.

Sheep flocks

Goat herds

Farms with intensive or semi-intensive management

2-year average	
Variables	Regression coefficient (± standard error)
Relative humidity at 2 m ($p = 0.044$)	0.009 ± 0.004

Farms with semi-extensive or extensive management

10-year average	
Variables	Regression coefficient (± standard error)
Maximum temperature at 2 m ($p = 0.010$)	–7.302 ± 2.812
Minimum temperature at 2 m ($p = 0.010$)	–7.302 ± 2.813
Temperature range at 2 m ($p = 0.010$)	–7.302 ± 2.812

Table S16. Results of multivariable analysis of climate conditions for associations with optional vaccination against paratuberculosis in 444 small ruminant farms in Greece.

Sheep flocks

2-year average	
Variables	Regression coefficient (± standard error)
Temperature at 2 m (p = 0.046)	–0.074 ± 0.038

Goat herds

Farms with intensive or semi-intensive management

10-year average	
Variables	Regression coefficient (± standard error)
Minimum temperature at 2 m (p = 0.0001)	–0.018 ± 0.004

Farms with semi-extensive or extensive management

10-year average	
Variables	Regression coefficient (± standard error)
Maximum temperature at 2 m (p = 0.048)	–0.016 ± 0.008

Table S17. Results of multivariable analysis of climate conditions for associations with optional vaccination against pneumonia in 444 small ruminant farms in Greece.

Sheep flocks

10-year average		2-year average	
Variables	Regression coefficient (± standard error)	Variables	Regression coefficient (± standard error)
Maximum temperature at 2 m (p = 0.0008)	0.028 ± 0.008	Relative humidity at 2 m (p = 0.0002)	–0.053 ± 0.014
Relative humidity at 2 m (p = 0.007)	0.041 ± 0.015	Maximum temperature at 2 m (p = 0.0018)	–0.026 ± 0.008

Goat herds

10-year average		2-year average	
Variables	Regression coefficient (± standard error)	Variables	Regression coefficient (± standard error)
Relative humidity at 2 m (p = 0.0002)	0.133 ± 0.035	Relative humidity at 2 m (p = 0.002)	–0.093 ± 0.029
Minimum temperature at 2 m (p = 0.003)	0.093 ± 0.030		
Temperature range at 2 m (p = 0.003)	0.080 ± 0.026		

Farms with intensive or semi-intensive management

10-year average		2-year average	
Variables	Regression coefficient (± standard error)	Variables	Regression coefficient (± standard error)
Temperature of Earth skin (p = 0.044)	–0.302 ± 0.149	Relative humidity at 2 m (p = 0.018)	–0.064 ± 0.027
		Maximum temperature at 2 m (p = 0.045)	–0.016 ± 0.008

Farms with semi-extensive or extensive management

10-year average		2-year average	
Variables	Regression coefficient (± standard error)	Variables	Regression coefficient (± standard error)
Relative humidity at 2 m (*p* = 0.014)	0.039 ± 0.016	Relative humidity at 2 m (*p* = 0.0004)	0.055 ± 0.015

Table S18. Results of multivariable analysis of climate conditions for associations with optional vaccination against staphylococcal mastitis in 444 small ruminant farms in Greece.

Sheep flocks

10-year average		2-year average	
Variables	Regression coefficient (± standard error)	Variables	Regression coefficient (± standard error)
Wind speed at 10 m ($p = 0.013$)	–0.078 ± 0.031	Wind speed at 10 m ($p = 0.010$)	–0.246 ± 0.095

Goat herds

2-year average	
Variables	Regression coefficient (± standard error)
Wind speed at 10 m ($p = 0.044$)	–0.224 ± 0.110

Farms with intensive or semi-intensive management

Farms with semi-extensive or extensive management

10-year average		2-year average	
Variables	Regression coefficient (± standard error)	Variables	Regression coefficient (± standard error)
Temperature of Earth skin ($p = 0.009$)	0.355 ± 0.134	Temperature at 2 m ($p = 0.002$)	–0.393 ± 0.127
Wind speed at 10 m ($p = 0.010$)	–0.203 ± 0.078	Temperature of Earth skin ($p = 0.003$)	0.370 ± 0.121
		Precipitation ($p = 0.014$)	0.193 ± 0.078
		Wind speed at 10 m ($p = 0.019$)	–0.174 ± 0.074

Table S19. Results of univariable analysis of climate conditions (correlation coefficient, *r*) for associations with the total number of optional vaccinations in 444 small ruminant farms in Greece.

Overall

10-year average	2-year average	*p*
**Temperature of Earth skin (°C)**		
–0.203	–0.213	< 0.0001 / < 0.0001
**Temperature at 2 m (°C)**		
–0.199	–0.203	< 0.0001 / < 0.0001
**Maximum temperature at 2 m (°C)**		
0.151	0.124	0.001 / 0.009
**Minimum temperature at 2 m (°C)**		
–0.179	–0.184	0.0001 / < 0.0001
**Temperature range at 2 m (°C)**		
0.233	0.220	< 0.0001 / < 0.0001
**Relative humidity at 2 m (%)**		
–0.063	–0.037	0.19 / 0.44
**Precipitation (mm)**		
0.094	0.110	0.048 / 0.020
**Wind speed at 10 m (m s^{-1})**		
–0.245	–0.247	< 0.0001 / < 0.0001

Sheep flocks

10-year average	2-year average	*p*
**Temperature of Earth skin (°C)**		
–0.224	–0.234	< 0.0001 / < 0.0001
**Temperature at 2 m (°C)**		
–0.207	–0.220	0.0002 / < 0.0001
**Maximum temperature at 2 m (°C)**		
0.136	0.109	0.14 / 0.049
**Minimum temperature at 2 m (°C)**		
–0.196	–0.200	0.0004 / 0.0003
**Temperature range at 2 m (°C)**		
0.247	0.232	< 0.0001 / 0.06

**Relative humidity at 2 m (%)**		
–0.070	–0.039	0.21 / 0.48
**Precipitation (mm)**		
0.094	0.122	0.09 / 0.028
**Wind speed at 10 m (m s^{-1})**		
–0.263	–0.264	< 0.0001 / < 0.0001

Goat herds

10-year average	2-year average	*p*
**Temperature of Earth skin (°C)**		
–0.156	–0.167	0.09 / 0.07
**Temperature at 2 m (°C)**		
–0.152	–0.166	0.10 / 0.07
**Maximum temperature at 2 m (°C)**		
0.230	0.210	0.012 / 0.022
**Minimum temperature at 2 m (°C)**		
–0.144	–0.148	0.12 / 0.11
**Temperature range at 2 m (°C)**		
0.198	0.192	0.031 / 0.036
**Relative humidity at 2 m (%)**		
–0.036	–0.027	0.70 / 0.77
**Precipitation (mm)**		
0.093	0.078	0.31 / 0.40
**Wind speed at 10 m (m s^{-1})**		
–0.198	–0.204	0.031 / 0.026

Farms with intensive or semi-intensive management

10-year average	2-year average	*p*
**Temperature of Earth skin (°C)**		
–0.038	–0.040	0.56 / 0.54
**Temperature at 2 m (°C)**		
–0.039	–0.043	0.55 / 0.51

**Maximum temperature at 2 m (^{o}C)**		
0.057	0.049	0.39 / 0.46
**Minimum temperature at 2 m (^{o}C)**		
–0.039	–0.030	0.55 / 0.65
**Temperature range at 2 m (^{o}C)**		
0.085	0.065	0.19 / 0.32
**Relative humidity at 2 m (%)**		
0.032	0.040	0.63 / 0.54
**Precipitation (mm)**		
–0.076	–0.011	0.25 / 0.87
**Wind speed at 10 m (m s^{-1})**		
–0.198	–0.072	0.002 / 0.27

Farms with semi-extensive or extensive management

10-year average	2-year average	***p***
**Temperature of Earth skin (^{o}C)**		
–0.228	–0.247	0.0009 / 0.0003
**Temperature at 2 m (^{o}C)**		
–0.220	–0.243	0.001 / 0.0004
**Maximum temperature at 2 m (^{o}C)**		
0.244	0.195	0.0004 / 0.005
**Minimum temperature at 2 m (^{o}C)**		
–0.182	0.191	0.008 / 0.005
**Temperature range at 2 m (^{o}C)**		
0.223	0.213	0.001 / 0.002
**Relative humidity at 2 m (%)**		
0.033	0.023	0.63 / 0.74
**Precipitation (mm)**		
0.188	0.255	0.006 / 0.0002
**Wind speed at 10 m (m s^{-1})**		
–0.246	–0.250	0.0003 / 0.0003

1. xxx / zzz: p-value for comparison between farms in which vaccination was or was not performed for 10-year average / 2-year average

Table S20. Results of multivariable analysis of climate conditions for associations with the total number of optional vaccinations in 444 small ruminant farms in Greece.

Overall

10-year average		2-year average	
Variables	Regression coefficient (± standard error)	Variables	Regression coefficient (± standard error)
Relative humidity at 2 m ($p < 0.0001$)	–0.369 ± 0.220	Temperature at 2 m ($p < 0.0001$)	–0.428 ± 0.108
Minimum temperature at 2 m ($p < 0.0001$)	–0.204 ± 0.041	Wind speed at 10 m ($p < 0.0001$)	–0.603 ± 0.144
Wind speed at 10 m ($p = 0.0002$)	–0.878 ± 0.231	Minimum temperature at 2 m ($p < 0.0001$)	–0.206 ± 0.050
Maximum temperature at 2 m ($p = 0.0004$)	–0.074 ± 0.021	Maximum temperature at 2 m ($p = 0.004$)	–0.071 ± 0.024
Temperature range at 2 m ($p = 0.002$)	–0.117 ± 0.038		

Sheep flocks

10-year average		2-year average	
Variables	Regression coefficient (± standard error)	Variables	Regression coefficient (± standard error)
Minimum temperature at 2 m ($p = 0.0003$)	0.261 ± 0.073	Minimum temperature at 2 m ($p = 0.0005$)	–0.246 ± 0.095
Temperature of Earth skin ($p = 0.0006$)	–0.512 ± 0.148	Temperature of Earth skin ($p = 0.0006$)	–0.448 ± 0.129
Maximum temperature at 2 m ($p = 0.002$)	–0.092 ± 0.030	Wind speed at 10 m ($p = 0.004$)	–0.472 ± 0.163
		Maximum temperature at 2 m ($p = 0.004$)	–0.076 ± 0.029

Goat herds

10-year average	
Variables	Regression coefficient (± standard error)
Maximum temperature at 2 m ($p = 0.033$)	0.323 ± 0.150

Farms with intensive or semi-intensive management

Farms with semi-extensive or extensive management

10-year average		2-year average	
Variables	Regression coefficient (± standard error)	Variables	Regression coefficient (± standard error)
Maximum temperature at 2 m ($p < 0.0001$)	0.482 ± 0.102	Minimum temperature at 2 m ($p < 0.0001$)	0.225 ± 0.064
Temperature at 2 m ($p < 0.0001$)	–0.707 ± 0.164	Temperature at 2 m ($p < 0.0001$)	–0.447 ± 0.129
Temperature range at 2 m ($p < 0.0001$)	–0.291 ± 0.071	Wind speed at 10 m ($p = 0.004$)	–0.472 ± 0.163
Precipitation ($p = 0.008$)	0.697 ± 0.259	Maximum temperature at 2 m ($p = 0.009$)	0.076 ± 0.029

Figure S2. Scatter plots of the total number of optional vaccinations versus the maximum temperature at 2 m (red dots) and the minimum temperature at 2 m (blue dots) in 444 small ruminant farms in Greece (dotted lines are respective trendlines).

(a) 10-year dataset

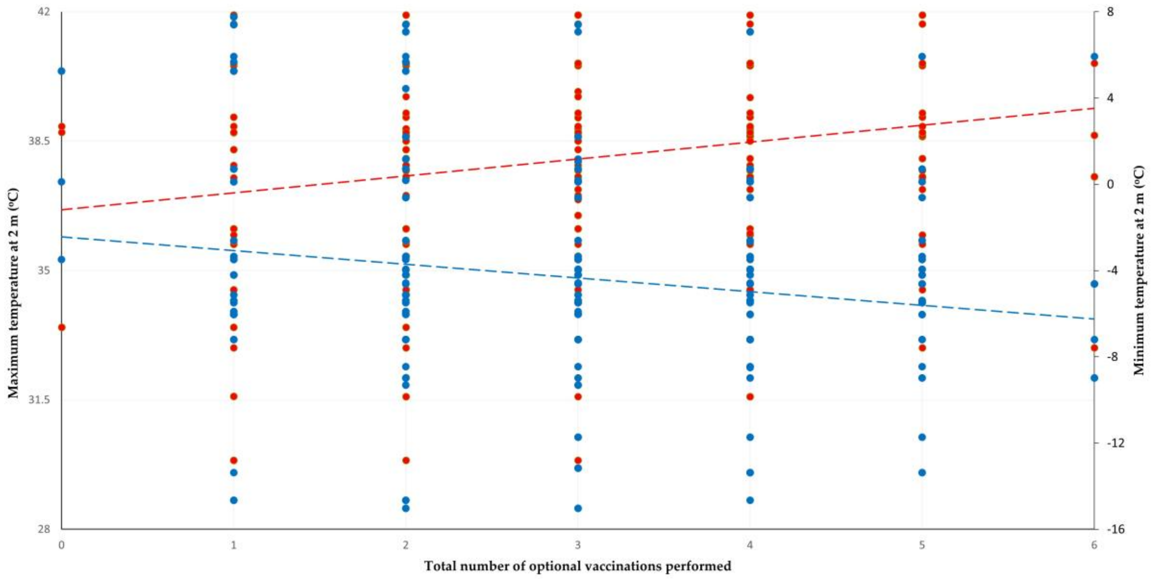

(b) 2-year dataset

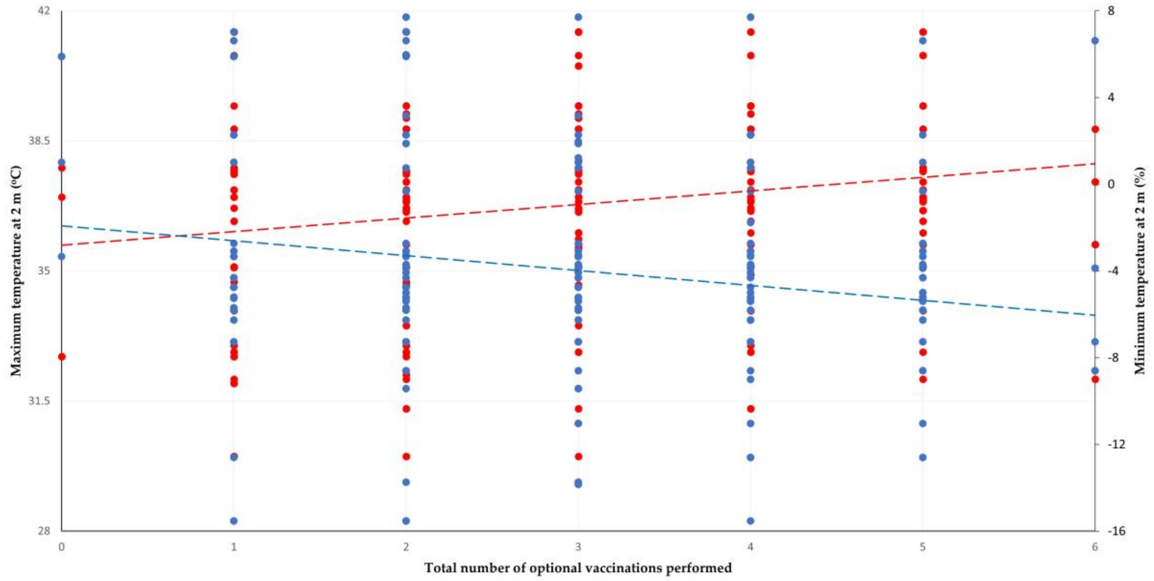

Table S21. Results of multivariable analysis for significant climatic, management-related or human resources-related parameters associated with optional vaccination against chlamydial abortion in 444 small ruminant farms in Greece.

Parameters	Odds Ratio [1]	*p*-value
Sheep flocks – 10-year average		
Age of newborns when taken away from dam		0.0003
≤ 40 days (53.8% [2])	reference	-
41–60 days (35.5%)	2.123 (1.315–3.428)	0.002
> 60 days (16.7%)	5.833 (2.258–15.067)	0.0003
Daily number of milking sessions		0.001
One (0.0%)	5.133 (0.201–131.421)	0.32
Two (34.8%)	3.229 (1.803–5.785)	0.0001
Three (63.3%)	reference	-
Wind speed at 10 m		0.026
per unit change	0.932 ± 1.032	0.026
Sheep flocks – 2-year average		
Age of newborns when taken away from dam		0.0006
≤ 40 days (53.8%)	reference	-
41–60 days (35.5%)	2.123 (1.315–3.428)	0.002
> 60 days (16.7%)	5.833 (2.258–15.067)	0.0003
Daily number of milking sessions		0.0009
One (0.0%)	5.133 (0.201–131.421)	0.32
Two (34.8%)	3.229 (1.803–5.785)	0.0001
Three (63.3%)	reference	-
Wind speed at 10 m		0.036
per unit change	0.936 ± 1.032	0.036
Goat herds – 10-year average		
No parameter emerged as significant		
Goat herds – 2-year average		
Average age of culling female animals		0.001
≤ 6.5 years (52.0%)	reference	-
> 6.5 years (18.8%)	4.667 (2.056 – 10.593)	0.0002
Daily period spent by farmer at the farm		0.002
≤ 8 hours (17.9%)	2.744 (0.954 – 7.890)	0.06
> 8 hours (37.4%)	reference	-
Daily number of milking sessions		0.031
One (25.0%)	18.000 (0.812 – 399.177)	0.07
Two (29.6%)	14.500 (1.648 – 123.188)	0.016
Three (85.7%)	reference	-
Breed of animals in the farm		0.032
Crossbreeds (38.9%)	0.971 (0.326 – 2.895)	0.957
Imported breeds (46.7%)	reference	-
Local breeds (20.0%)	2.471 (1.050 – 5.814)	0.038
Age of newborns when taken away from dam		0.037
≤ 40 days (57.7%)	reference	-
41 – 60 days (27.3%)	3.636 (1.308 – 10.110)	0.013
> 60 days (24.5%)	4.205 (1.524 – 11.597)	0.006
Farms with intensive or semi-intensive management – 10-year average		
Daily number of milking sessions		< 0.0001
Two (70/176 = 39.8%)	4.291 (2.080–8.850)	0.0001
Three (34/46 = 73.9%)	reference	-
Farms with intensive or semi-intensive management – 2-year average		
Daily number of milking sessions		< 0.0001
Two (70/176 = 39.8%)	4.291 (2.080–8.850)	0.0001
Three (34/46 = 73.9%)	reference	-

Farms with semi-extensive or extensive management – 10-year average		
Wind speed at 10 m		0.0007

per unit change	0.714 ± 1.103	0.0007
Temperature at 2 m		0.006
per unit change	1.110 ± 1.038	0.006
Relative humidity at 2 m		0.014
per unit change	1.070 ± 1.028	0.014
Age of newborns when taken away from dam		0.037
≤ 40 days (57.8%)	reference	-
41 – 60 days (23.0%)	4.579 (2.193 – 9.560)	0.0001
> 60 days (20.3%)	5.368 (2.297 – 12.548)	0.0001
Average age of culling female animals		0.040
≤ 6.5 years (38.8%)	reference	-
> 7 years (16.1%)	3.291 (1.707 – 6.345)	0.0004
Farms with semi-extensive or extensive management – 2-year average		
Precipitation		0.007
per unit change	–1.225 ± 0.075	0.007
Average age of culling female animals		0.010
≤ 6.5 years (38.8%)	reference	-
> 7 years (16.1%)	3.291 (1.707 – 6.345)	0.0004
Age of newborns when taken away from dam		0.031
≤ 40 days (57.8%)	reference	-
41 – 60 days (23.0%)	4.579 (2.193 – 9.560)	0.0001
> 60 days (20.3%)	5.368 (2.297 – 12.548)	0.0001

1 Odds ratios calculated against the highest prevalence associations of the variable.
2 Proportion of farms in which the event of interest was seen among those in which the studied variable prevailed.

Table S22. Results of multivariable analysis for significant climatic, management-related or human resources-related parameters associated with optional vaccination against clostridial infections in 444 small ruminant farms in Greece.

Parameters	Odds Ratio [1]	*p*-value
	Sheep flocks – 10-year average	
Temperature of Earth skin		< 0.0001
per unit change	0.739 ± 1.047	< 0.0001
Temperature at 2 m		< 0.0001
per unit change	1.411 ± 1.054	< 0.0001
Relative humidity at 2 m		0.0001
per unit change	1.026 ± 1.007	0.0001
Precipitation		0.006
per unit change	–0.911 ± 1.034	0.006
Family tradition in farming		0.014
Yes (97.9% [2])	reference	-
No (92.9%)	3.551 (0.853–14.779)	0.08
Age of farmers		0.048
≤50 years (99.0%)	reference	-
>50 years (94.5%)	5.641 (1.153–27.598)	0.033
	Sheep flocks – 2-year average	
Temperature of Earth skin		< 0.0001
per unit change	0.806 ± 1.073	< 0.0001
Temperature at 2 m		< 0.0001
per unit change	1.280 ± 0.043	< 0.0001
Relative humidity at 2 m		0.005
per unit change	1.015 ± 0.005	0.0001
Family tradition in farming		0.016
Yes (97.9%)	reference	-
No (92.9%)	3.551 (0.853–14.779)	0.08
	Goat herds – 10-year average	
Family tradition in farming		0.011
Yes (100.0%)	reference	-
No (93.8%)	20.032 (0.781–513.999)	0.07
	Goat herds – 2-year average	
Family tradition in farming		0.011
Yes (100.0%)	reference	-
No (93.8%)	20.032 (0.781–513.999)	0.07
	Farms with intensive or semi-intensive management – 10-year average	
	No parameter emerged as significant	
	Farms with intensive or semi-intensive management – 2-year average	
	No parameter emerged as significant	
	Farms with semi-extensive or extensive management – 10-year average	
Temperature of Earth skin		< 0.0001
per unit change	0.752 ± 1.051	< 0.0001
Temperature at 2 m		< 0.0001
per unit change	1.343 ± 1.053	< 0.0001
Family tradition in farming		0.0002
Yes (54.2%)	reference	-
No (25.0%)	22.111 (4.814-101.558)	0.0001
Wind speed at 10 m		0.002
per unit change	1.102 ± 0.031	0.002
Age of farmers		0.026
≤50 years (54.2%%)	reference	-
>50 years (25.0%)	3.551 (0.701-17.993)	0.13

Farms with semi-extensive or extensive management – 2-year average
No parameter emerged as significant

1 Odds ratios calculated against the highest prevalence associations of the variable.

2 Proportion of farms in which the event of interest was seen among those in which the studied variable prevailed.

Table S23. Results of multivariable analysis for significant climatic, management-related or human resources-related parameters associated with optional vaccination against contagious agalactia in 444 small ruminant farms in Greece.

Parameters	Odds Ratio [1]	*p*-value
Sheep flocks – 10-year average		
Wind speed at 10 m		0.0002
per unit change	0.853 ± 0.043	0.0002
Management system applied in farms		0.001
Intensive (81.8% [2])	reference	-
Semi-intensive (63.6%)	2.579 (1.113–5.972)	0.027
Semi-extensive (50.0%)	4.500 (1.927–10.509)	0.0005
Extensive (12.0%)	33.000 (7.905–137.760)	< 0.0001
Collaboration with a veterinarian		0.032
Yes (61.1%)	reference	-
No (31.0%)	3.508 (1.748–7.040)	0.0004
Sheep flocks – 2-year average		
Wind speed at 10 m		< 0.0001
per unit change	0.840 ± 0.043	0.0001
Management system applied in farms		0.001
Intensive (81.8% [2])	reference	-
Semi-intensive (63.6%)	2.579 (1.113–5.972)	0.027
Semi-extensive (50.0%)	4.500 (1.927–10.509)	0.0005
Extensive (12.0%)	33.000 (7.905–137.760)	< 0.0001
Relative humidity at 2 m		0.030
per unit change	1.034 ± 0.015	0.030
Collaboration with a veterinarian		0.045
Yes (61.1%)	reference	-
No (31.0%)	3.508 (1.748–7.040)	0.0004
Goat herds – 10-year average		
Use of laboratory diagnostic examinations in samples of milk		0.002
Yes (88.0%)	reference	
No (45.7%)	8.698 (2.436–31.056)	0.0009
Wind speed at 10 m		0.012
per unit change	0.885 ± 1046	0.012
Collaboration with a veterinarian		0.032
Yes (60.8%)	reference	-
No (17.6%)	7.233 (1.954–26.776)	0.003
Goat herds – 2-year average		
Use of laboratory diagnostic examinations in samples of milk		0.002
Yes (88.0%)	reference	
No (45.7%)	8.698 (2.436–31.056)	0.0009
Wind speed at 10 m		0.006
per unit change	0.884 ± 1.045	0.006
Collaboration with a veterinarian		0.012
Yes (60.8%)	reference	-
No (17.6%)	7.233 (1.954–26.776)	0.003
Farms with intensive or semi-intensive management – 10-year average		
Wind speed at 10 m		0.0002
per unit change	0.830 ± 1.051	0.0002
Duration of dry-period		0.047
≤2 months (75.8%)	Reference	-
>2 months (61.0%)	1.936 (1.067-3.512)	0.030

Farms with intensive or semi-intensive management – 2-year average		
Wind speed at 10 m		0.0006
per unit change	0.842 ± 1.051	0.0006
Use of laboratory diagnostic examinations in samples of milk		0.019
Yes (83.6%)	reference	-
No (61.7%)	3.176 (1.457-6.925)	0.004
Farms with semi-extensive or extensive management – 10-year average		
Collaboration with a veterinarian		< 0.0001
Yes (53.3%)	reference	
No (12.2%)	8.229 (3.088-21.929)	< 0.0001
Temperature at 2 m		0.002
per unit change	0.722 ± 1.085	0.002
Maximum temperature at 2 m		0.008
per unit change	–1.100 ± 1.036	0.008
Use of laboratory diagnostic examinations in samples of milk		0.015
Yes (70.0%)	reference	-
No (40.7%)	3.405 (1.628-7.124)	0.001
Temperature range at 2 m		0.015
per unit change	–0.942 ± 1.025	0.015
Farms with semi-extensive or extensive management – 2-year average		
Collaboration with a veterinarian		0.0001
Yes (53.3%)	reference	
No (12.2%)	8.229 (3.088-21.929)	< 0.0001
Temperature at 2 m		0.002
per unit change	0.856 ± 0.050	0.002
Use of laboratory diagnostic examinations in samples of milk		0.015
Yes (70.0%)	reference	-
No (40.7%)	3.405 (1.628-7.124)	0.001
Maximum temperature at 2 m		0.027
per unit change	–1.074 ± 1.033	0.027
Temperature range at 2 m		0.036
per unit change	–0.958 ± 1.021	0.036

1 Odds ratios calculated against the highest prevalence associations of the variable.

2 Proportion of farms in which the event of interest was seen among those in which the studied variable prevailed.

Table S24. Results of multivariable analysis for significant climatic, management-related or human resources-related parameters associated with optional vaccination against contagious ecthyma in 444 small ruminant farms in Greece.

Parameters	Odds Ratio [1]	*p*-value
Sheep flocks – 10-year average		
Minimum temperature at 2 m		0.0003
per unit change	0.991 ± 1.003	0.0003
Maximum temperature at 2 m		0.031
per unit change	0.997 ± 1.001	0.031
Temperature range at 2 m		0.032
per unit change	–0.996 ± 1.002	0.032
Farmer's general education		0.033
Primary education (3.5% [2])	reference	-
Secondary or post-secondary education (0.4%)	8.109 (0.722–91.063)	0.09
Tertiary education (0.0%)	4.009 (0.188–85.667)	0.37
Sheep flocks – 2-year average		
Minimum temperature at 2 m		0.0007
per unit change	0.997 ± 1.001	0.0007
Temperature range at 2 m		0.039
per unit change	–0.996 ± 1.002	0.039
Maximum temperature at 2 m		0.049
per unit change	0.991 ± 1.003	0.049
Goat herds – 10-year average		
No parameter emerged as significant		
Goat herds – 2-year average		
No parameter emerged as significant		
Farms with intensive or semi-intensive management – 10-year average		
No parameter emerged as significant		
Farms with intensive or semi-intensive management – 2-year average		
Temperature at 2 m		0.0002
per unit change	0.985 ± 1.004	0.0002
Farms with semi-extensive or extensive management – 10-year average		
Farmer's general education		0.025
Primary education (4.8%)	reference	-
Secondary or post-secondary education (0.0%)	19.074 (0.898–405.208)	0.06
Tertiary education (0.0%)	3.148 (0.145–68.265)	0.47
Farms with semi-extensive or extensive management – 2-year average		
Farmer's general education		0.016
Primary education (4.8%)	reference	-
Secondary or post-secondary education (0.0%)	19.074 (0.898–405.208)	0.06
Tertiary education (0.0%)	3.148 (0.145–68.265)	0.47
Maximum temperature at 2 m		0.049
per unit change	0.019 ± 7.367	0.049
Temperature range at 2 m		0.049
per unit change	–52.269 ± 7.367	0.049
Minimum temperature at 2 m		0.049
per unit change	52.199 ± 7.368	0.049

1 Odds ratios calculated against the highest prevalence associations of the variable.
2 Proportion of farms in which the event of interest was seen among those in which the studied variable prevailed.

Table S25. Results of multivariable analysis for significant climatic, management-related or human resources-related parameters associated with optional vaccination against foot-rot in 444 small ruminant farms in Greece.

Parameters	Odds Ratio [1]	*p*-value
Sheep flocks – 10-year average		
No parameter emerged as significant		
Sheep flocks – 2-year average		
No parameter emerged as significant		
Goat herds – 10-year average		
No parameter emerged as significant		
Goat herds – 2-year average		
No parameter emerged as significant		
Farms with intensive or semi-intensive management – 10-year average		
Farmer's general education		0.022
Primary education (0.0% [2])	3.580 (0.180-71.361)	0.40
Secondary or post-secondary education (0.8%)	5.492 (0.600-53.875)	0.14
Tertiary education (4.4%)	reference	-
Farms with intensive or semi-intensive management – 2-year average		
Relative humidity at 2 m		0.044
per unit change	–1.009 ± 0.004	0.044
Farmer's general education		0.047
Primary education (0.0%)	3.580 (0.180-71.361)	0.40
Secondary or post-secondary education (0.8%)	5.492 (0.600-53.875)	0.14
Tertiary education (4.4%)	reference	-
Farms with semi-extensive or extensive management – 10-year average		
Maximum temperature at 2 m		0.014
per unit change	–1046.24 ± 16.481	0.014
Temperature range at 2 m		0.014
per unit change	–0.001 ± 16.483	0.014
Minimum temperature at 2 m		0.049
per unit change	0.001 ± 16.486	0.049
Farms with semi-extensive or extensive management – 2-year average		
Farmer's general education		0.030
Primary education (0.0%)		
Secondary or post-secondary education (0.0%)		
Tertiary education (1.7%)	reference	-

1 Odds ratios calculated against the highest prevalence associations of the variable.
2 Proportion of farms in which the event of interest was seen among those in which the studied variable prevailed.

Table S26. Results of multivariable analysis for significant climatic, management-related or human resources-related parameters associated with optional vaccination against paratuberculosis in 444 small ruminant farms in Greece.

Parameters	Odds Ratio [1]	*p*-value
Sheep flocks – 10-year average		
No parameter emerged as significant		
Sheep flocks – 2-year average		
No parameter emerged as significant		
Goat herds – 10-year average		
No parameter emerged as significant		
Goat herds – 2-year average		
No parameter emerged as significant		
Farms with intensive or semi-intensive management – 10-year average		
Minimum temperature at 2 m		0.0002
per unit change	0.984 ± 1.004	0.0002
Farms with intensive or semi-intensive management – 2-year average		
No parameter emerged as significant		
Farms with semi-extensive or extensive management – 10-year average		
No parameter emerged as significant		
Farms with semi-extensive or extensive management – 2-year average		
No parameter emerged as significant		

1 Odds ratios calculated against the highest prevalence associations of the variable.

Table S27. Results of multivariable analysis for significant climatic, management-related or human resources-related parameters associated with optional vaccination against pneumonia in 444 small ruminant farms in Greece.

Parameters	Odds Ratio [1]	*p*-value
	Sheep flocks – 10-year average	
Maximum temperature at 2 m		0.002
per unit change	–1.024 ± 0.008	0.002
Routine administration of antibiotics to newborns		0.007
Yes (58.6% [2])	reference	-
No (40.4%)	2.086 (1.219–3.571)	0.007
Relative humidity at 2 m		0.018
per unit change	–1.036 ± 0.015	0.018
Daily period spent by farmer at the farm		0.029
≤8 h (34.9%)	1.813 (1.111–2.959)	0.017
>8 h (48.7%)	reference	-
	Sheep flocks – 2-year average	
Relative humidity at 2 m		0.0005
per unit change	–1.050 ± 1.014	0.0005
Maximum temperature at 2 m		0.0007
per unit change	–1.027 ± 0.008	0.0007
Routine administration of antibiotics to newborns		0.007
Yes (58.6%)	reference	-
No (40.4%)	2.086 (1.219–3.571)	0.007
Daily period spent by farmer at the farm		0.030
≤8 h (34.9%)	1.813 (1.111–2.959)	0.017
>8 h (48.7%)	reference	-
	Goat herds – 10-year average	
Relative humidity at 2 m		0.001
per unit change	–1.124 ± 0.035	0.001
Minimum temperature at 2 m		0.010
per unit change	–1.084 ± 1.031	0.010
Temperature range at 2 m		0.014
per unit change	1.068 ± 0.026	0.014
	Goat herds – 2-year average	
	No parameter emerged as significant	
	Farms with intensive or semi-intensive management – 10-year average	
Relative humidity at 2 m		0.003
per unit change	–1.057 ± 1.018	0.003
Daily period spent by farmer at the farm		0.047
≤8 h (34.9%)	1.813 (1.111–2.959)	0.017
>8 h (48.7%)	reference	-
	Farms with intensive or semi-intensive management – 2-year average	
Relative humidity at 2 m		0.024
per unit change	–1.041 ± 0.018	0.024
	Farms with semi-extensive or extensive management – 10-year average	
Relative humidity at 2 m		0.028
per unit change	–1.036 ± 0.016	0.028
	Farms with semi-extensive or extensive management – 2-year average	
Relative humidity at 2 m		0.001
per unit change	–1.052 ± 0.016	0.001

1 Odds ratios calculated against the highest prevalence associations of the variable.
2 Proportion of farms in which the event of interest was seen among those in which the studied variable prevailed.

Table S28. Results of multivariable analysis for significant climatic, management-related or human resources-related parameters associated with optional vaccination against staphylococcal mastitis in 444 small ruminant farms in Greece.

Parameters	Odds Ratio [1]	*p*-value
Sheep flocks – 10-year average		
Use of laboratory diagnostic examinations in samples of milk		0.005
Yes (55.7% [2])	reference	-
No (34.1%)	2.429 (1.419–4.161)	0.001
Collaboration with a veterinarian		0.017
Yes (42.0%)	reference	-
No (16.7%)	3.628 (1.558–8.447)	0.003
Sheep flocks – 2-year average		
Use of laboratory diagnostic examinations in samples of milk		0.007
Yes (55.7% [2])	reference	-
No (34.1%)	2.429 (1.419–4.161)	0.001
Collaboration with a veterinarian		0.010
Yes (42.0%)	reference	-
No (16.7%)	3.628 (1.558–8.447)	0.003
Goat herds – 10-year average		
Type of milking mode		0.002
Machine-milking (40.9%)	reference	-
Hand-milking (13.2%)	4.550 (1.787–11.581)	0.002
Daily period spent by farmer at the farm		0.006
≤8 h (17.9%)	2.152 (0.743–6.228)	0.16
>8 h (31.9%)	reference	-
Daily number of milking sessions		0.016
One (0/4 = 0.0%)	19.800 (0.744–527.292)	0.07
Two (29/108 = 26.9%)	6.810 (1.251–37.063)	0.027
Three (5/7 = 71.4%)	reference	-
Goat herds – 2-year average		
Type of milking mode		0.002
Machine-milking (40.9%)	reference	-
Hand-milking (13.2%)	4.550 (1.787–11.581)	0.002
Daily period spent by farmer at the farm		0.006
≤8 h (17.9%)	2.152 (0.743–6.228)	0.16
>8 h (31.9%)	reference	-
Daily number of milking sessions		0.016
One (0/4 = 0.0%)	19.800 (0.744–527.292)	0.07
Two (29/108 = 26.9%)	6.810 (1.251–37.063)	0.027
Three (5/7 = 71.4%)	reference	-
Farms with intensive or semi-intensive management – 10-year average		
Use of laboratory diagnostic examinations in samples of milk		0.026
Yes (55.7% [2])	reference	-
No (34.1%)	2.429 (1.419–4.161)	0.001
Farms with intensive or semi-intensive management – 2-year average		
Use of laboratory diagnostic examinations in samples of milk		0.026
Yes (55.7% [2])	reference	-
No (34.1%)	2.429 (1.419–4.161)	0.001
Farms with semi-extensive or extensive management – 10-year average		
Type of milking mode		0.0002
Machine-milking (39.5%)	reference	-
Hand-milking (11.8%)	4.896 (2.418–9.915)	< 0.0001

Farms with semi-extensive or extensive management – 2-year average		
Type of milking mode		< 0.0001
Machine-milking (39.5%)	reference	-
Hand-milking (11.8%)	4.896 (2.418–9.915)	< 0.0001
Temperature at 2 m		0.0009
per unit change	0.666 ± 1.129	0.0009
Temperature of Earth skin		0.001
per unit change	1.471 ± 1.122	0.001
Wind speed at 10 m		0.025
per unit change	0.852 ± 1.074	0.025
Precipitation		0.030
per unit change	–1.180 ± 1.078	0.030

1 Odds ratios calculated against the highest prevalence associations of the variable.

2 Proportion of farms in which the event of interest was seen among those in which the studied variable prevailed.

Table S29. Summary of multivariable analyses for outcomes regarding optional vaccinations in small ruminant farms in Greece.

Outcome	Farm	10-year average		2-year average	
		Variable	*p*	Variable	*p*
Vaccination against chlamydial abortion	S [1]	Age of newborns when taken away from dam	0.0003	Age of newborns when taken away from dam	0.0006
		Daily number of milking sessions	0.001	Daily number of milking sessions	0.0009
		Wind speed at 10 m	0.026	Wind speed at 10 m	0.036
	G [1]	--		Average age of culling female animals	0.001
				Daily period spent by farmer at the farm	0.002
				Daily number of milking sessions	0.031
				Breed of animals in the farm	0.032
				Age of newborns when taken away from dam	0.037
	I & s-I [1]	Daily number of milking sessions	< 0.0001	Daily number of milking sessions	< 0.0001
	s-E & E [1]	Wind speed at 10 m	0.0007	Precipitation	0.007
		Temperature at 2 m	0.006	Average age of culling female animals	0.010
		Relative humidity at 2 m	0.014	Age of newborns when taken away from dam	0.031
		Age of newborns when taken away from dam	0.037		
		Average age of culling female animals	0.040		
Vaccination against clostridial infections	S	Temperature of Earth skin	< 0.0001	Temperature of Earth skin	< 0.0001
		Temperature at 2 m	< 0.0001	Temperature at 2 m	< 0.0001
		Relative humidity at 2 m	0.0001	Relative humidity at 2 m	0.005
		Precipitation	0.006	Family tradition in farming	0.016
		Family tradition in farming	0.014		
		Age of farmers	0.048		
	G	Family tradition in farming	0.011	Family tradition in farming	0.011
	I & s-I	--		--	
	s-E & E	Temperature of Earth skin	< 0.0001		
		Temperature at 2 m	< 0.0001		
		Family tradition in farming	0.0002		
		Wind speed at 10 m	0.002		
		Age of farmers	0.026		

Vaccination against contagious agalactia	S	Wind speed at 10 m	0.0002	Wind speed at 10 m	< 0.0001
		Management system applied in farms	0.001	Management system applied in farms	0.001
		Collaboration with a veterinarian	0.032	Relative humidity at 2 m	0.030
				Collaboration with a veterinarian	0.045
	G	Use of laboratory diagnostic examinations in samples of milk	0.002	Use of laboratory diagnostic examinations in samples of milk	0.002
		Wind speed at 10 m	0.012	Wind speed at 10 m	0.006
		Collaboration with a veterinarian	0.032	Collaboration with a veterinarian	0.012
	I & s-I	Wind speed at 10 m	0.0002	Wind speed at 10 m	0.0006
		Duration of dry-period	0.047	Use of laboratory diagnostic examinations in samples of milk	0.019
	s-E & E	Collaboration with a veterinarian	< 0.0001	Collaboration with a veterinarian	0.0001
		Temperature at 2 m	0.002	Temperature at 2 m	0.002
		Maximum temperature at 2 m	0.008	Use of laboratory diagnostic examinations in samples of milk	0.015
		Use of laboratory diagnostic examinations in samples of milk	0.015	Maximum temperature at 2 m	0.027
		Temperature range at 2 m	0.015	Temperature range at 2 m	0.036
Vaccination against contagious ecthyma	S	Minimum temperature at 2 m	0.0003	Minimum temperature at 2 m	0.0007
		Maximum temperature at 2 m	0.031	Temperature range at 2 m	0.039
		Temperature range at 2 m	0.032	Maximum temperature at 2 m	0.049
		Farmer's general education	0.033		
	G	--		--	
	I & s-I	--		Temperature at 2 m	0.0002
	s-E & E	Farmer's general education	0.025	Farmer's general education	0.016
				Maximum temperature at 2 m	0.049
				Temperature range at 2 m	0.049
				Minimum temperature at 2 m	0.049
Vaccination against foot-rot	S	--		--	
	G	--		--	
	I & s-I	Farmer's general education	0.022	Relative humidity at 2 m	0.044
				Farmer's general education	0.047
	s-E & E	Maximum temperature at 2 m	0.014	Farmer's general education	0.030
		Temperature range at 2 m	0.014		
		Minimum temperature at 2 m	0.049		
Vaccination against paratuberculosis	S	--		--	
	G	--		--	
	I & s-I	Minimum temperature at 2 m	0.0002	--	
	s-E & E	--		--	

Vaccination against pneumonia	S	Maximum temperature at 2 m	0.002	Relative humidity at 2m	0.0005
		Routine administration of antibiotics to newborns	0.007	Maximum temperature at 2 m	0.0007
		Relative humidity at 2 m	0.018	Routine administration of antibiotics to newborns	0.007
		Daily period spent by farmer at the farm	0.029	Daily period spent by farmer at the farm	0.030
	G	Relative humidity at 2 m	0.001	--	
		Minimum temperature at 2 m	0.010		
		Temperature range at 2 m	0.014		
	I & s-I	Relative humidity at 2 m	0.003	Relative humidity at 2 m	0.024
		Daily period spent by farmer at the farm	0.047		
	s-E & E	Relative humidity at 2 m	0.028	Relative humidity at 2 m	0.001
Vaccination against staphylococcal mastitis	S	Use of laboratory diagnostic examinations in samples of milk	0.005	Use of laboratory diagnostic examinations in samples of milk	0.007
		Collaboration with a veterinarian	0.017	Collaboration with a veterinarian	0.010
	G	Type of milking mode	0.002	Type of milking mode	0.002
		Daily period spent by farmer at the farm	0.006	Daily period spent by farmer at the farm	0.006
		Daily number of milking sessions	0.016	Daily number of milking sessions	0.016
	I & s-I	Use of laboratory diagnostic examinations in samples of milk	0.026	Use of laboratory diagnostic examinations in samples of milk	0.026
	s-E & E	Type of milking mode	0.0002	Type of milking mode	<0.0001
				Temperature at 2 m	0.0009
				Temperature of Earth skin	0.001
				Wind speed at 10 m	0.025
				Precipitation	0.030

1 S: sheep, G: goat, I: intensive, E: extensive, s: semi.

Table S30. Results of multivariable analysis for significant climatic, management-related or human resources-related parameters associated with total number of optional vaccinations in 444 small ruminant farms in Greece.

Parameters	Odds Ratio [1]	*p*-value
Sheep flocks – 10-year average		
Management system applied in farms		0.004
per unit change	0.766 ± 1.099	0.004
Temperature of Earth skin		0.006
per unit change	0.690 ± 1.120	0.006
Temperature at 2 m		0.007
per unit change	1.076 ± 1.030	0.007
Age of newborns when taken away from dam		0.010
per unit change	0.760 ± 1.112	0.010
Minimum temperature at 2 m		0.013
per unit change	1.188 ± 0.069	0.013
Routine administration of antibiotics to newborns		0.015
per unit change	0.667 ± 1.179	0.015
Daily number of milking sessions		0.016
per unit change	1.512 ± 1.186	0.016
Use of laboratory diagnostic examinations in samples of milk		0.019
per unit change	1.469 ± 1.177	0.019
Sheep flocks – 2-year average		
Temperature of Earth skin		0.001
per unit change	0.672 ± 1.129	0.001
Management system applied in farms		0.002
per unit change	0.775 ± 1.095	0.002
Minimum temperature at 2 m		0.004
per unit change	1.185 ± 1.060	0.004
Temperature at 2 m		0.004
per unit change	1.076 ± 1.027	0.004
Age of newborns when taken away from dam		0.008
per unit change	0.754 ± 1.110	0.008
Daily number of milking sessions		0.008
per unit change	1.571 ± 1.186	0.008
Routine administration of antibiotics to newborns		0.010
per unit change	0.654 ± 1.177	0.010
Use of laboratory diagnostic examinations in samples of milk		0.017
per unit change	1.477 ± 1.176	0.017
Goat herds – 10-year average		
Maximum temperature at 2 m		0.006
per unit change	1.143 ± 1.049	0.006
Goat herds – 2-year average		
No parameter emerged as significant		
Farms with intensive or semi-intensive management – 10-year average		
Daily number of milking sessions		0.003
per unit change	1.802 ± 1.220	0.003
Farms with intensive or semi-intensive management – 2-year average		
Daily number of milking sessions		0.003
per unit change	1.802 ± 1.220	0.003
Farms with semi-extensive or extensive management – 10-year average		
Maximum temperature at 2 m		0.0004
per unit change	1.425 ± 1.103	0.0004
Use of laboratory diagnostic examinations in samples of milk		0.0006
per unit change	2.156 ± 1.245	0.0006

Temperature at 2 m		0.001
per unit change	0.596 ± 1.171	0.001
Collaboration with a veterinarian		0.009
per unit change	0.557 ± 1.248	0.009
Daily period spent by farmer at the farm		0.015
per unit change	1.615 ± 1.216	0.015
Temperature range at 2 m		0.018
per unit change	0.806 ± 1.070	0.018
Farms with semi-extensive or extensive management – 2-year average		
Minimum temperature at 2 m		0.0002
per unit change	1.338 ± 1.079	0.0002
Temperature at 2 m		0.0002
per unit change	0.591 ± 1.148	0.0002
Use of laboratory diagnostic examinations in samples of milk		0.0007
per unit change	2.116 ± 1.243	0.0007
Collaboration with a veterinarian		0.002
per unit change	0.513 ± 1.242	0.002
Wind speed at 10 m		0.010
per unit change	0.448 ± 1.271	0.010
Daily period spent by farmer at the farm		0.037
per unit change	1.506 ± 1.216	0.037
Age of farmers		0.043
per unit change	0.711 ± 1.182	0.043

1 Odds ratios calculated against the highest prevalence associations of the variable.
